# Identifying and Quantifying Heterogeneity in High Content Analysis: Application of Heterogeneity Indices to Drug Discovery

**DOI:** 10.1371/journal.pone.0102678

**Published:** 2014-07-18

**Authors:** Albert H. Gough, Ning Chen, Tong Ying Shun, Timothy R. Lezon, Robert C. Boltz, Celeste E. Reese, Jacob Wagner, Lawrence A. Vernetti, Jennifer R. Grandis, Adrian V. Lee, Andrew M. Stern, Mark E. Schurdak, D. Lansing Taylor

**Affiliations:** 1 Drug Discovery Institute, University of Pittsburgh, Pittsburgh, Pennsylvania, United States of America; 2 Department of Computational & Systems Biology, University of Pittsburgh, Pittsburgh, Pennsylvania, United States of America; 3 University of Pittsburgh Cancer Institute, University of Pittsburgh, Pittsburgh, Pennsylvania, United States of America; 4 Department of Otolaryngology, University of Pittsburgh, Pittsburgh, Pennsylvania, United States of America; 5 Department of Pharmacology and Chemical Biology, University of Pittsburgh, Pittsburgh, Pennsylvania, United States of America; Stanford University School of Medicine, United States of America

## Abstract

One of the greatest challenges in biomedical research, drug discovery and diagnostics is understanding how seemingly identical cells can respond differently to perturbagens including drugs for disease treatment. Although heterogeneity has become an accepted characteristic of a population of cells, in drug discovery it is not routinely evaluated or reported. The standard practice for cell-based, high content assays has been to assume a normal distribution and to report a well-to-well average value with a standard deviation. To address this important issue we sought to define a method that could be readily implemented to identify, quantify and characterize heterogeneity in cellular and small organism assays to guide decisions during drug discovery and experimental cell/tissue profiling. Our study revealed that heterogeneity can be effectively identified and quantified with three indices that indicate diversity, non-normality and percent outliers. The indices were evaluated using the induction and inhibition of STAT3 activation in five cell lines where the systems response including sample preparation and instrument performance were well characterized and controlled. These heterogeneity indices provide a standardized method that can easily be integrated into small and large scale screening or profiling projects to guide interpretation of the biology, as well as the development of therapeutics and diagnostics. Understanding the heterogeneity in the response to perturbagens will become a critical factor in designing strategies for the development of therapeutics including targeted polypharmacology.

## Introduction

One of the greatest challenges in drug discovery and development is understanding how seemingly identical cells respond differently to drug treatment [1]. In cancer, the prevalence of intra-tumor genetic and phenotypic heterogeneity, results from clonal evolution [2], [3], epigenetic plasticity[4] and variation in tumor microenvironments [5] and suggest that a single drug targeting a single driver is not likely to adequately control disease progression [6]. The complexity of the tumor microenvironment, which extends to stromal cells, including immune cells, may contribute significantly to the development of resistance to treatment [5]. Efforts to recapitulate the *in vivo* tumor microenvironment in physiologically relevant models will require analytical approaches that address the heterogeneity in the model [7], [8]. However, cellular heterogeneity is not limited to cancer cells, but is exhibited even in normal, clonal cell lines, and the impact of heterogeneity extends from basic biology to drug discovery and diagnostics [9]–[11].

It is now understood that there are multiple sources of heterogeneity in cell populations including both genetic and non-genetic factors. Genetic variation is well studied [4], [12], [13]. Non-genetic heterogeneity, also referred to as phenotypic heterogeneity, is variability of one or more phenotypes or traits within a clonal population [9]. Non-genetic heterogeneity has been organized into a hierarchy of dichotomies starting with extrinsic versus intrinsic factors [9]. Variation in extrinsic factors results from variation in the cellular microenvironment. Intrinsic heterogeneity arises from intracellular factors, even in a uniform environment, and can be further subdivided into macro- and micro-heterogeneity [9]. The former refers to the variability in one or more cell traits that result in discrete phenotypes and the latter to the apparently continuous random variation within a single phenotype. It is widely accepted that non-genetic heterogeneity plays an important biological role in cell behaviors such as cell fate decision in stem cells, development and cellular physiology [9]–[11]. It is also of increasing interest in tumor diagnostics, therapeutics and disease management, as well as drug discovery and development [14]–[17].

A major opportunity in drug discovery is to apply a quantitative systems pharmacology (QSP) approach to modulating the biochemical networks that are involved in disease, in contrast to identifying and validating a single molecular target up front [18]–[20]. High Content Analysis (HCA) [21], [22], flow cytometry [23], single cell genomics [24] and other “phenotypic” methods provide the capability to measure multiple biomarkers in large numbers of individual cells. In particular, HCA can be used to profile individual cells within tissues and small animal models, as well as in 2D and 3D arrays of cells [15], [25]. However, it has been standard practice in HCA to reduce the detailed cellular data to a population average (well average) that is intended to characterize the overall response of the cells, assuming a normal distribution [26].

The plate-to-plate and the day-to-day variabilities of HCA measurements are usually characterized by the Z’ factor or the strictly standardized mean difference (SSMD) [26]–[29]. These metrics assume a normal distribution of the well average data [30]. However, there has not been a similar effort in HCA to address phenotypic heterogeneity in a simple, standard and quantitative manner amenable to medium to high throughput screening. There have been multiple studies in which cellular heterogeneity was evaluated and characterized. For example, classifiers were trained to identify subpopulations based on collections of phenotypic features. In some cases the subpopulations were characterized by the median and interquartile range [31]. In addition, an analysis based on visual analytics combining parallel-coordinate plots, used for a visual assessment of the high-dimensional dependencies, and nonlinear support vector machines, for the quantification of heterogeneity, has also been demonstrated [32]. A heterogeneity scoring approach (HetMap) was designed to visualize the heterogeneity within an individual patient's breast tissue based on immunohistochemistry in the context of a patient population [33]. Furthermore, analytical tools such as Kolmogorov-Smirnov (KS) statistics, machine learning, and univariate and multivariate analyses have been applied to analyze perturbations in cells with drugs and siRNA [34]–[43]. These analytical tools have been valuable for characterizing heterogeneity and demonstrating the value of heterogeneity analysis in drug discovery, pathway analysis and diagnostics, but are not optimal for routine evaluation of large-scale screens or profiles.

The goal of the present paper is to describe a method for the analysis of cellular heterogeneity in cellular phenotypes that includes: developing a set of “indices” to identify, quantify and characterize heterogeneity in a way that it can be easily included in all screening and cellular profiling; as well as to demonstrate an optimal data representation to visualize the full range of heterogeneity in the data when it is identified. We use heterogeneity in the activation of STAT3 as a model system for developing and testing indices and show how the heterogeneity indices can be used in high throughput biology and drug discovery to quantify, compare and flag studies in which: 1) there is a high degree of variability in the cellular responses, 2) results suggest there is more than one subpopulation, or 3) there are more than the expected number of outliers. This important information will also be essential to interpreting cellular responses in multiplexed, 2D and 3D screens, as well as within more complex microenvironments *in vivo* and *in vitro,* in physiologically relevant disease and organ models, as well as patient samples.

## Materials and Methods

### Cell culture

Cal33 human head and neck squamous cell carcinoma (HNSCC) cells [44], [45] were kindly provided by Dr. Gerard Milano (University of Nice, Nice, France). The cell line was maintained in Dulbecco's modified Eagle's medium (Life Technologies) supplemented with 10% fetal bovine serum (Gemini Bio-Products), 100 U/ml penicillin and 100 µg/ml streptomycin (HyClone). MCF-7 and MDA-MB-468 human breast carcinoma cells [ATCC cell lines obtained from Dr. Adrian Lee, University of Pittsburgh] were cultured in DMEM Glutamax media (Life Technologies) supplemented with 10% FBS (Gemini Bio-Products), 100 U/ml penicillin and 100 µg/ml streptomycin (HyClone). MCF-10A human breast cells [ATCC cell line obtained from Dr. Adrian Lee] were cultured in DMEM F12 media (Life Technologies) supplemented with 5% Horse Serum (Life Technologies), 10 µg/ml Insulin (Sigma-Aldrich), 20 ng/ml Epidermal Growth Factor (Sigma-Aldrich), 20 ng/ml Cholera Toxin (Sigma-Aldrich), 500 ng/ml Hydrocortisone (Sigma-Aldrich), and 1% Penicillin/Streptomycin (Life Technologies). All cell lines were maintained in humidified incubators at 37°C with 5% CO_2_.

### Stimulation and inhibition of STAT3 phosphorylation

Cal33 cells were plated in collagen-coated 384-well plates (Greiner Bio-One) at 2000 cells/well to reach 50% confluence on the day of fixation. The cells were incubated at 37°C for 24 hours followed by serum deprivation for another 24 hours. For stimulation of STAT3 phosphorylation, human recombinant interleukin-6 (IL-6) and Oncostatin M (OSM) (R&D Systems) were added in 2-fold or 2.4-fold serial dilution for 10 final concentrations descending from 200 ng/ml or 50 ng/ml, respectively. For the time course of cellular response to stimulation, the cells were incubated with cytokines at 37°C for 15, 30, 45, 60 and 120 minutes before fixation. For inhibition of STAT3 phosphorylation, Pyridone-6 (Calbiochem) was added in 10-point 3-fold serial dilution for final concentrations descending from 5 µM. Stattic (Sigma-Aldrich) was added in 10-point 3-fold serial dilution for final concentrations descending from 50 µM. After 3 hours incubation with the inhibitors at 37°C, cells were stimulated with 50 ng/ml of IL-6 for 15 minutes (peak induction time) before fixation. Each treatment was performed in triplicate. Each experiment was repeated at least 3 times. The assays were optimized for cell density, cytokine dose and treatment time with their robustness validated using the Z’ factor [27]. Each plate included 16 positive and 16 negative control wells from which the Z’ was calculated (ranged from 0.5 to 0.8).

### Immunofluorescence labeling

Cells were treated with cytokines with or without incubation with inhibitors, then fixed in plates for 30 minutes at room temperature with 3.7% Formaldehyde in PBS, 2 µg/ml Hoechst 33342 (Life Technologies) for nuclear staining, and then permeabilized on ice with 95% MeOH in PBS for 30 minutes. Permeabilized cells were washed and incubated with 0.1% Tween-20 in PBS at 4°C overnight. Cells were labeled for 1 hour with a 1∶800 dilution of mouse anti-pY705 STAT3 antibody (BD Biosciences) followed by a 1 hour incubation with a 1∶300 dilution of the Alexa Fluor 647-donkey anti-mouse AffiniPure secondary antibody (Jackson ImmunoResearch) prior to high content imaging. The fixation procedure and antibody titrations were optimized individually.

### High Content Analysis

Labeled cells were imaged with an ArrayScan VTI (ThermoFisher -Cellomics) using a 10X (0.45NA) objective, a stable LED illumination source with excitations of 386/23 nm and 650/13 nm, and a multiband emission filter with transmission at 440/40 nm and 700/60 nm for Hoechst (Ch1) and AlexaFluor 647 (Ch3), respectively. Image correction for non-uniformity of the field of the VTI was accomplished using an Opera Adjustment Plate (PerkinElmer) which contains uniform dye solutions as targets for reference image collection. Images were corrected using the VTI acquisition software and analyzed using the Compartmental Analysis Bioapplication (Thermo Fisher- Cellomics). Briefly, the Hoechst nuclear images were segmented using an isodata threshold. The DNA content was measured as the Bioapplication feature ObjectTotalIntenCh1 for the selected nuclear region (‘circ’). The ‘circ’ regions were copied to channel 3 where it was used to construct a ‘ring’ starting 1 pixel out from the ‘circ’ and 3 pixels wide. The STAT3 phosphorylation was measured using the Bioapplication feature CircRingAvgIntenDiffCh3. Images of four fields (typically ranges from 2000 to 2500 cells total) were acquired for each well. The pixel average nucleus-cytoplasm difference (CircRingAvgIntenDiffCh3) in pSTAT3 fluorescence intensity (referred as “Relative Activity” in the plots) was calculated for each cell in all four fields. Cell level data were retrieved from the Cellomics Store Database (ThermoFisher) into Spotfire (TIBCO) using SQL queries and plotted for dose and time dependent responses. Experimental metadata were merged into Spotfire using an Excel (Microsoft) template and the combined data was analyzed to generate a ‘histo-box plot’ as described below.

### Flow Cytometry

Cell and Bead Standards: Flow cytometry standards were used to establish the resolution and linearity of the high content imaging relative to that of a flow cytometer. In this study we used Becton Dickinson 2 µm beads (DNA QC Particles 349523, Becton Dickinson).

Flow Cytometry: The LSR II (Becton Dickinson) was configured with UV-355 nm, Violet-404 nm, Blue-488 nm and red-633 excitations. The pulse emission areas were collected with the following filters: Hoechst 33342–450/50 nm, Fluorescein – 530/30 nm, Cy3 – 610/20 nm, Cy5 – 660/20 nm and Cy7 – 780/60 nm. To establish that high content imaging was capable of acquiring accurate DNA histograms, Cal33 cells were run on both the LSR II flow cytometer and the ArrayScan VTI High Content imager (ThermoFisher). The cells were trypsinized, fixed, stained with Hoechst 33342 (Life Technologies) at 2 µg/ml. The flow cytometry sample was run as a suspension of 2×10^6^ cells/ml at a rate of ∼100 cells/sec. The data were then analyzed with FlowJo 7.6.5 (TreeStar) or exported as FCS 3 format for cell cycle analysis in ModFit (Verity Software House). For high content imaging, the Cal33 cell suspension was spun down on a 96 well microplate. The data were exported to text files that were converted to FCS format with Text to FCS Software 1.2.1 and then analyzed identically to the flow cytometry data.

### Visualization of subpopulation distributions

A modification of a standard ‘box plot’ was generated in Spotfire to visualize the distributions of cellular responses and identify heterogeneity in those cell populations. The ‘box’ in a box plot represents the extent of the central 50% of the population but gives no indication of the distribution of those values. Spotfire includes an option in the ‘Appearance’ settings for the box plot to overlay the distribution (histogram) which we used to create the visualization we refer to as a ‘histo-box plot’. Similar plots, such as violin plots [46] and bean plots [47], can be created in R [48], Matlab (MathWorks) and other applications. The histo-box plot was used to analyze experimental data from the High Content Screening assays. Parameters characterizing the distribution such as the interquartile range (IQR), lower and upper inner fences and percent outliers were calculated. Log scaling of histo-box plots was done by applying the ‘Log10’ function in Spotfire to the average CircRingAvgIntenDiffCh3 values and is referred as ‘Log (Relative Activity)’ in the plots.

### Data analysis

Statistical measures used for heterogeneity analysis:
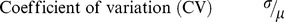
(1)

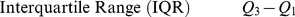
(2)

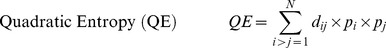
(3)

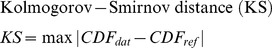
(4)where σ is the standard deviation (SD), µ is the mean, *Q*_n_ is the n-th quartile, *N* is the number of data values, *d* is a linear matrix of intensity differences between data points *i* and *j*, *p* is the probability distribution of data points, and *CDF_dat_* and *CDF_ref_* are the cumulative distribution functions of the data and a reference distribution, respectively. Quadratic entropy was calculated as a summation over 64 equally spaced bins spanning the range from -34 to 1441 to minimize the finite size effects associated with the binning scheme. Scalar statistic *KS* values were computed for each sample distribution using the MATLAB (MathWorks) function ‘kstest()’ and a reference normal distribution generated as a Gaussian distribution: 
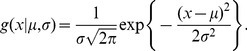
(5)with the mean *µ* and standard deviation *σ* of the measured sample distribution and *g* is the probability density about x.

## Results

### Characterization of heterogeneity in HCS

**Figure 1:**
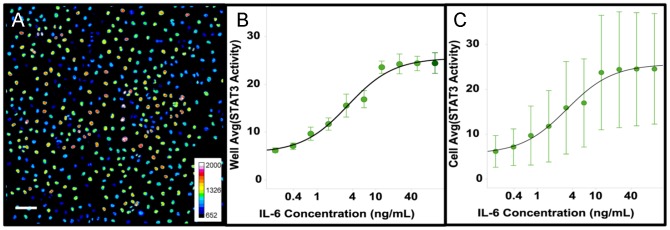
Heterogeneity in the activation STAT3 in Cal33 cells. Cal33 cells were treated with IL-6 (50 ng/ml) for 15 min. then fixed and labeled with an antibody to phospho-STAT3-Y705. A) Pseudocolor image of STAT3 activation shows a high degree of heterogeneity in the intensity of the Cy5-labeled secondary antibody (color scale at lower right indicates mapping of relative fluorescent intensities to colors). Scale bar is 100 um (lower left). B) The standard deviation of the well average STAT3 activity in replicate wells (EC50 = 3.3 ng/ml, error bars are ±1σ, N = 8) indicates the assay is highly reproducible despite the observed cellular heterogeneity (Z’ = 0.54) C) The standard deviation of the cellular STAT3 activity (error bars are ±1σ) indicates the high variability in the cell-to-cell STAT3 Activity consistent with the appearance of the image (A).

We optimized the assay and performed an HCS screen to measure the activation of the STAT3 signaling pathway in response to interleukin 6 (IL-6) and/or Oncostatin M (OSM) using an antibody against phospho-STAT3-Y705 [49]. The assay was optimized and validated for timing and duration of activation, as well as robustness in several cancer and normal cell lines (Figure S1). The induction of STAT3 by IL-6 in Cal33 cells exhibited a high level of cell-to-cell variation even at the optimal exposure time of 15 minutes and a dose that produced maximal activation (≥50 ng/ml IL-6). The variability in the fluorescence intensity of the nuclear localized, activated (phosphorylated) STAT3 was easily observed in the images (Figure 1A). Despite this cell to cell heterogeneity within each well, the assay was highly reproducible by standard criteria (a Z’≥0.5, and a signal-to-background >5) and exhibited a typical dose-response (Figure 1B). However, the reproducibility indicated in Figure 1B is a measure of the well-to-well reproducibility and does not give any indication of the cell-to-cell variability demonstrated by the large error bars (±1 SD) within a well (Figure 1C). Application of standard assay performance criteria like Z’ to the characterization of cell-to-cell reproducibility would result in a highly negative Z’, indicating high cell-to-cell variability, as we observed in Figure 1A and 1C, but no insight into the nature of the cellular heterogeneity. Clearly a different approach to characterizing cellular heterogeneity is needed as a complement to determining the Z’ and S/B for an assay.

**Figure 2:**
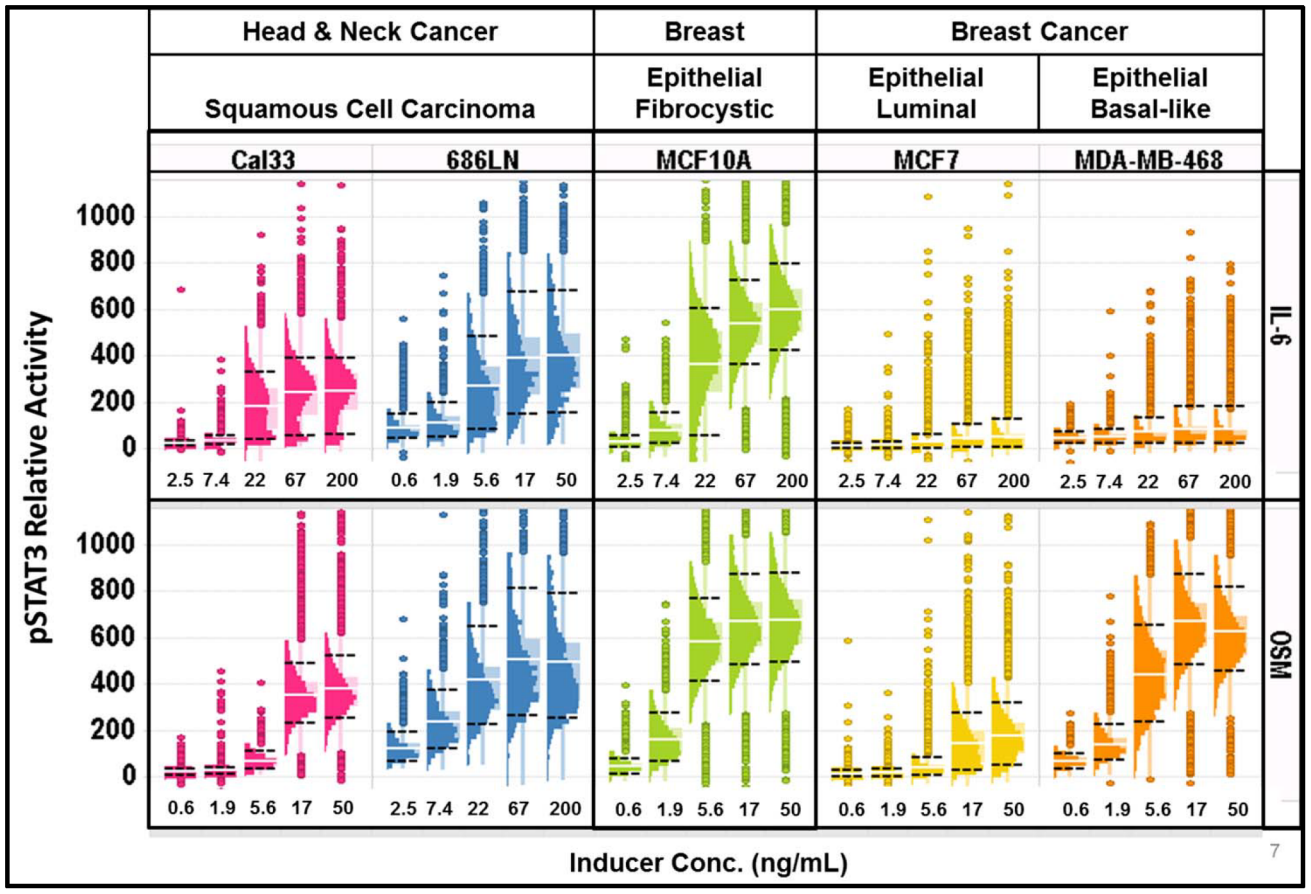
Variation in the cellular distributions of STAT3 activation by IL-6 and OSM in several cell types. Top series) Histo-box plots of the activation of STAT3 by IL-6 after 15 min exposure to IL-6 at the indicated concentrations in 2 HNSCC cell lines, 1 breast cell line and 2 breast cancer cell lines. Bottom series) The activation of STAT3 by OSM was measured at 15 min. in the same 5 cell lines as above. Note: 686LN cells were found to be much more sensitive to IL-6 and much less sensitive to OSM than the other cell lines, so the concentrations were adjusted appropriately.

To determine whether the high degree of variation in the level of STAT3 activation was unique to the Cal33 cell line, and/or activation by IL-6, we validated the assay on a panel of 5 cell lines and then compared the activation of STAT3 by 2 different cytokines, IL-6 and OSM. Figure 2 shows example distributions of STAT3 activation by 5 doses of IL-6 or OSM in the 5 cell lines (data provided as DataArchive S1). To visualize the distribution and statistical parameters of the cellular responses we used a standard box plot with an overlaid histogram that we refer to as a ‘histo-box plot’ (see Figure S2 for more details). The interquartile range (IQR, the ‘box’) extends from the 1^st^ quartile to the 3^rd^ quartile and comprises 50% of the data. The histogram extends from the lower inner fence (LIF) to the upper inner fence (UIF). Outliers, points outside the range from LIF to UIF (indicated as individual points on the plot), as well as reference indicators of the average (white line) and the 10^th^ and 90^th^ percentiles (black dashed lines) are presented in the plot. Note that although a standard box plot indicates the median of the distribution, here for reference we show the more commonly used assay parameter, the average value. Clearly the distributions of the activation of STAT3 vary widely between these cell types, and cytokines.

As illustrated in Figure 2, Cal33 cells exhibit a bimodal distribution in response to IL-6 and a more “normal” distribution (though with more outliers) in response to OSM, demonstrating that different signaling ligands can induce distinct patterns of heterogeneity in the same cell type. In contrast, 686LN and MCF-10A cells exhibit a similar distribution of responses to both IL-6 and OSM. The 686LN cells were ≈4-fold more sensitive to IL-6 and ≈2-fold less sensitive to OSM than either Cal33 or MCF-10A cells. The breast cancer cell lines, MCF-7 and MDA-MB-468, showed little or no response to IL-6, with an apparent dose-dependent increase in outliers, indicating that a small subset of the cells respond to IL-6 to the same extent as Cal33 or 686LN cells and with the same sensitivity as Cal33 cells. The breast cancer cells exhibit very different responses to OSM. The MDA-MB-468 cells response to OSM is similar to the MCF-10A cells, while the majority of MCF-7 cells respond over a range from seemingly non-responsive to low level responders with outliers exhibiting a high level response comparable to MCF-10A and MDA-MB-468. In summary, we observed differences in distributions between cell lines and between cytokines that varied from narrow to broad, bimodal vs. normal and variation in the number of outliers. The presence of macro-heterogeneity, micro-heterogeneity and a variable % of outliers were evident in these data.

To ensure that the observed heterogeneity was not due to instrumental or measurement variability (instrument systems response), we compared HCA measurements of standard fluorescent beads and DNA content in Cal33 cells with measurements of the same samples by flow cytometry (Table S1, Figure S3). In both cases the imaging CV was 2–3% higher than the flow cytometry CV, as expected, but still only about 5% for beads and about 8% for DNA content, well below the CV of ≈ 50% in Figure 1C. Therefore, instrumental systems response is not an explanation for the heterogeneity.

In this study, simple biological explanations for the heterogeneity observed in Figure 2 could include a dependence on cell cycle and/or expression level of the IL-6 receptor. We investigated the potential cell cycle dependence of the activation of STAT3 and found there was no correlation between cell cycle phase and STAT3 activation by IL-6 (Figure S4). Western blot analysis of the expression levels of IL-6 in the cell lines indicates that responsiveness is not directly correlated with total receptor expression (Figure S5). Determination of the molecular basis of the heterogeneity is an important challenge and is being pursued with a range of experimental approaches, including live cell, kinetic studies of STAT3 activation, but is beyond the scope of this investigation.

**Figure 3:**
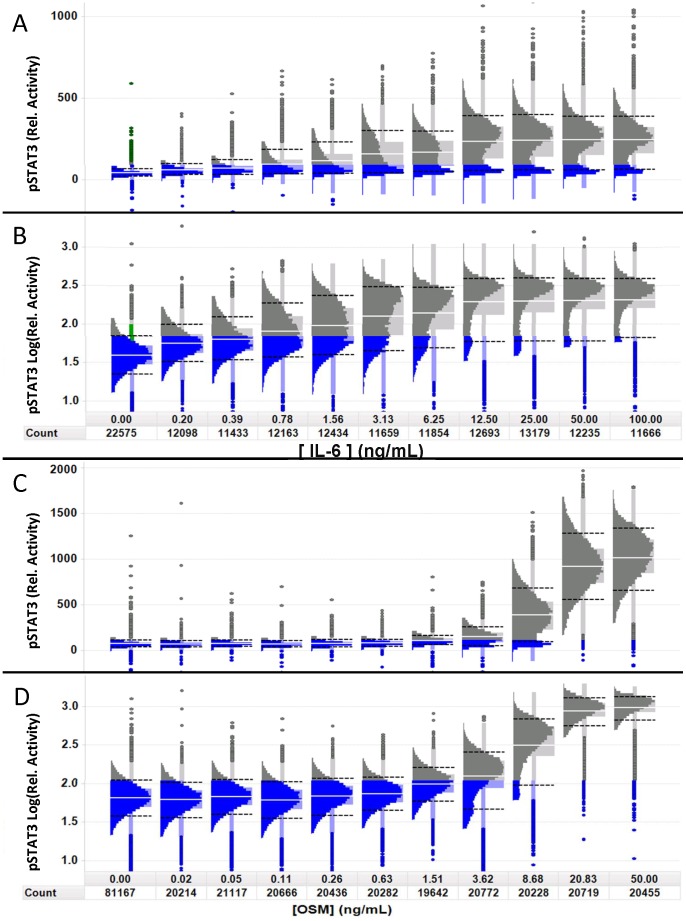
Visual analysis of phenotypic heterogeneity using the histo-box plot. Population distributions of STAT3 activity in Cal33 cells at the peak induction time of 15^th^ percentile of the untreated cells (left most histogram) are colored in blue to highlight the apparently non-responsive subpopulations. “Count” indicates the total number of cells measured. A) Linear-scaled dose-response distributions of STAT3 activity at the indicated concentrations of IL-6 show a persistent subpopulation of cells with a distribution comparable to the unstimulated cells. The well average EC50 = 3.3 ng/ml. B) Log-scaling of the same distributions in A shows that the CV of the responding cells (far right) is similar to the unstimulated cells (far left). C) Linear-scaled population distributions of STAT3 activation by OSM at the indicated concentrations also show a non-responding subpopulation at 8.68 ng/ml, but unlike IL-6 there are only a few outliers that are apparently non-responsive at 50 ng/ml and the responding cells appear to be more normally distributed. D) Log-scaling of the same distributions in C shows that the CV of the responding cells (far right) is similar to the unstimulated cells (far left).

Because the Cal33 cells exhibited a bimodal distribution in response to IL-6 and a more normal distribution in response to OSM, we decided to examine those dose-responses in more detail (Figure 3, DataArchive S1). STAT3 activation by IL-6 in Cal33 cells exhibited a bimodal distribution, with a ≥10% apparently non-responding subpopulation at all concentrations tested (Figure 3A and 3B). For comparison of distributions with different means, or potentially log-normal distributions [50], we also used a log-scaled histo-box plot (see Figure S2). Linear scale plots (Figure 3A & 3C) allow visualization of the intensity range, separation and size of the subpopulations. When plotted on a log scale (Figure 3B & 3D), the apparent width of a distribution is proportional to the linear CV, independent of the mean intensity, allowing direct comparison of the subpopulation CVs (micro-heterogeneity). The linear scaled histo-box plot exhibits a narrow distribution of cells at the unstimulated level of STAT3 intensity (highlighted in blue), with only a few outliers of activated cells (<2%). Upon activation with IL-6, there remains a distinct and persistent subpopulation (≈10% at the saturation level of stimulation-100 ng/ml) of apparently non-responding cells (highlighted in blue in Figure 3) and a heterogeneous population of responding cells. The log scaling (Figure 3B) showed that the CV of the distribution of the unstimulated cell population is about the same as that of the stimulated population (58% and 50% respectively). The presence of the 2 sub-populations, responding and apparently non-responding is an example of macro-heterogeneity. Macro-heterogeneity is distinguished from outliers in that outliers are singular observations that are well separated from the median, above the UIF (median +1.5*IPR) or below the LIF (median – 1.5*IPR), and of insufficient density to be detected above the background in a histogram. The limits of detecting a subpopulation will depend on the number and distribution of cells and may include subpopulation distributions with significant overlap. Multiplexed assays, looking at multiple parameters will assist in further defining the sub-populations [31].

Cal33 cells exhibited a different distribution of STAT3 activation in response to OSM. At the OSM saturation concentration, STAT3 activation was observed in essentially all the cells (Figure 3). The bimodal distribution was only apparent at intermediate OSM concentrations (3.62 ng/ml and 8.68 ng/ml) suggesting two subpopulations of cells, one activated at a lower dose of OSM, while the other responding at a higher dose, in contrast to the persistence of the IL-6 resistant subpopulation even at high doses of IL-6 (Figure 3C and D). Overall, OSM stimulation of Cal33 cells resulted in a higher degree of activation of STAT3 than stimulation with IL-6. To evaluate whether the cells that do not appear to respond to IL-6 stimulation were in fact capable of phosphorylating STAT3, we induced cells with combinations of IL-6 and OSM (Figure S6). Even at the lowest concentration of OSM tested (2.78 ng/ml) cells co-induced with any concentration of IL-6 did not exhibit a non-responding subpopulation. Furthermore, at this low concentration of OSM (2.78 ng/ml), OSM alone did not induce a STAT3 response, suggesting there may be some cooperativity between IL-6 and OSM activation of STAT3. This will be investigated in more detail in a future study.

Although the analysis of heterogeneity described above using the histo-box plot provides insights into functional responses, such interactive analysis is limited to relatively small numbers of experiments where throughput is not critical. For large scale profiling and particularly in compound screening, a method to identify and quantify heterogeneity is needed in order to compare large numbers of compounds, targets, or assays and to make decisions about next steps, efficiently.

### Selection and evaluation of the heterogeneity indices (HI's)

**Figure 4:**
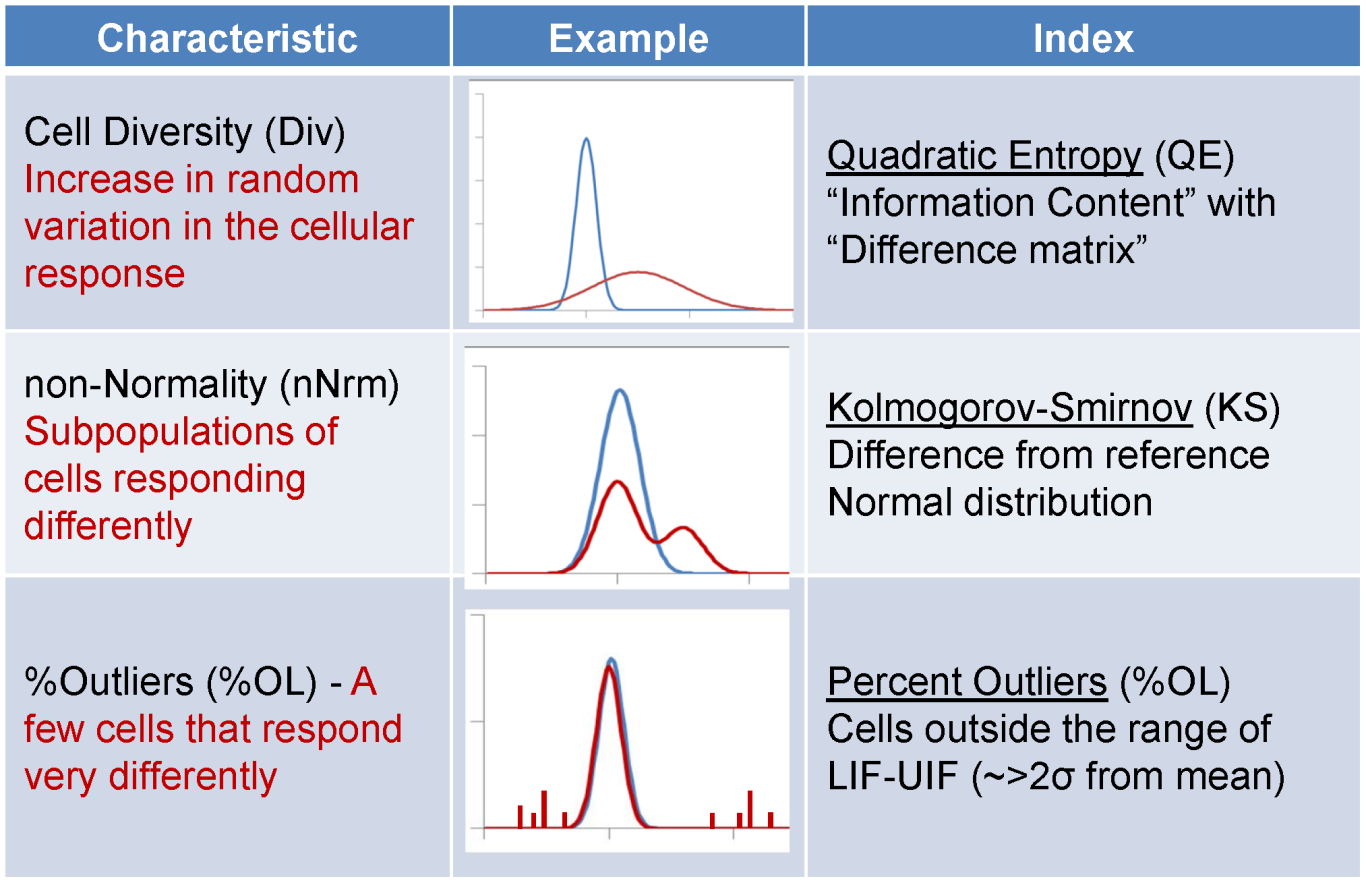
Three indices for characterizing cellular heterogeneity. Three indices that provide information about the distribution were chosen. Cell Diversity (DIV) characterizes the overall heterogeneity in the population without regard for the specific shape of the distribution, using the Quadratic Entropy, a metric that is sensitive to the spread of the distribution as well as the magnitude of the differences between phenotypes in the distribution. Non-Normality (nNRM) indicates deviation from a normal distribution, distinguishing between micro- and macro-heterogeneity. %Outliers (%OL) indicates the fraction of cells that respond differently than the majority.

**Figure 5:**
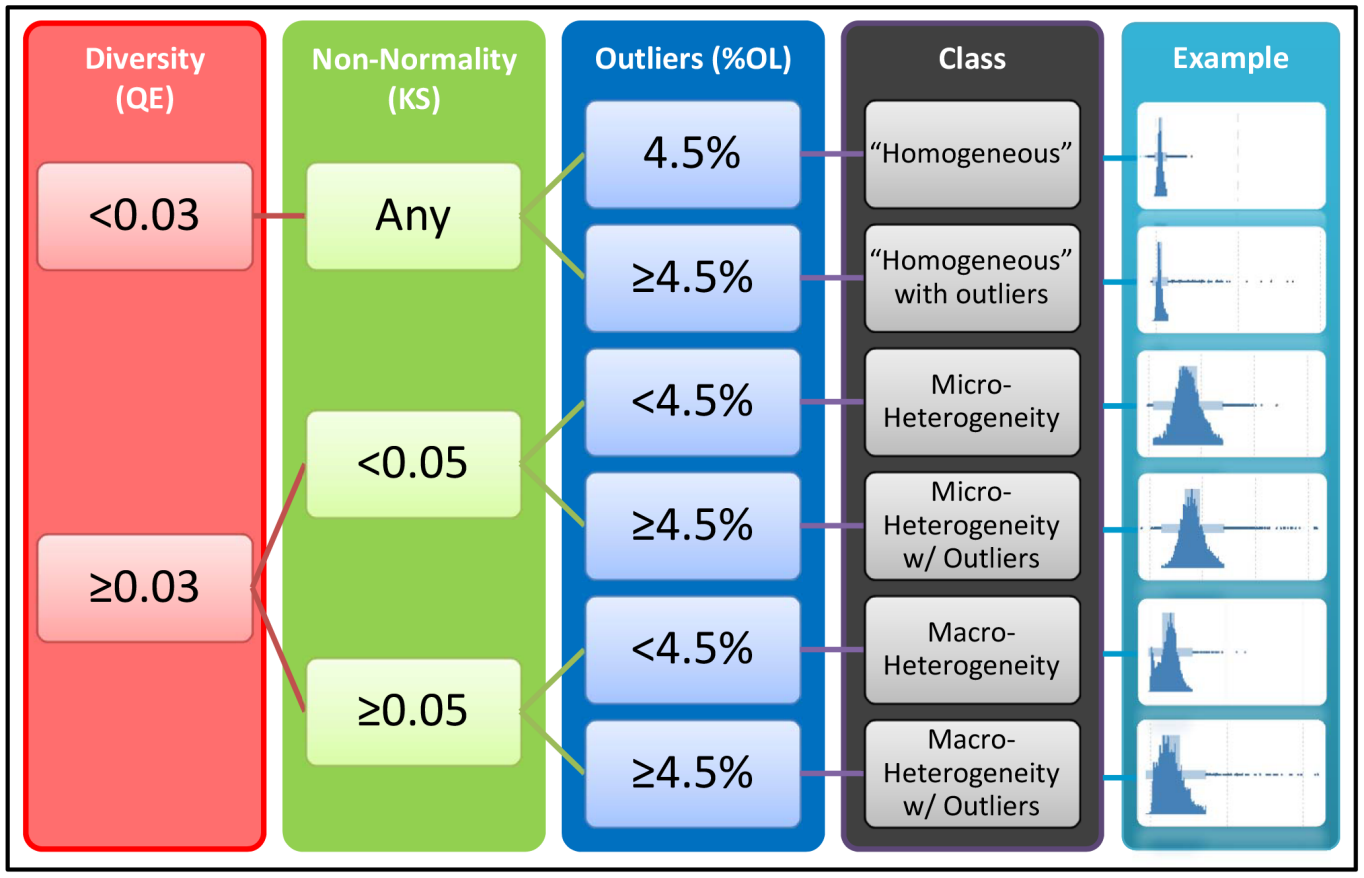
Decision tree for interpreting the Heterogeneity Indices. Using thresholds established for each index, DIV, nNRM and %OL, a binary decision tree can be used to characterize heterogeneity in a given sample. The thresholds for DIV (0.03) and nNRM (0.05) were selected as the mean +3 SD for each index in replicate negative control wells for Cal33 cells. The threshold for %OL (4.5%) is the percent outliers expected for a normal distribution.

**Figure 6:**
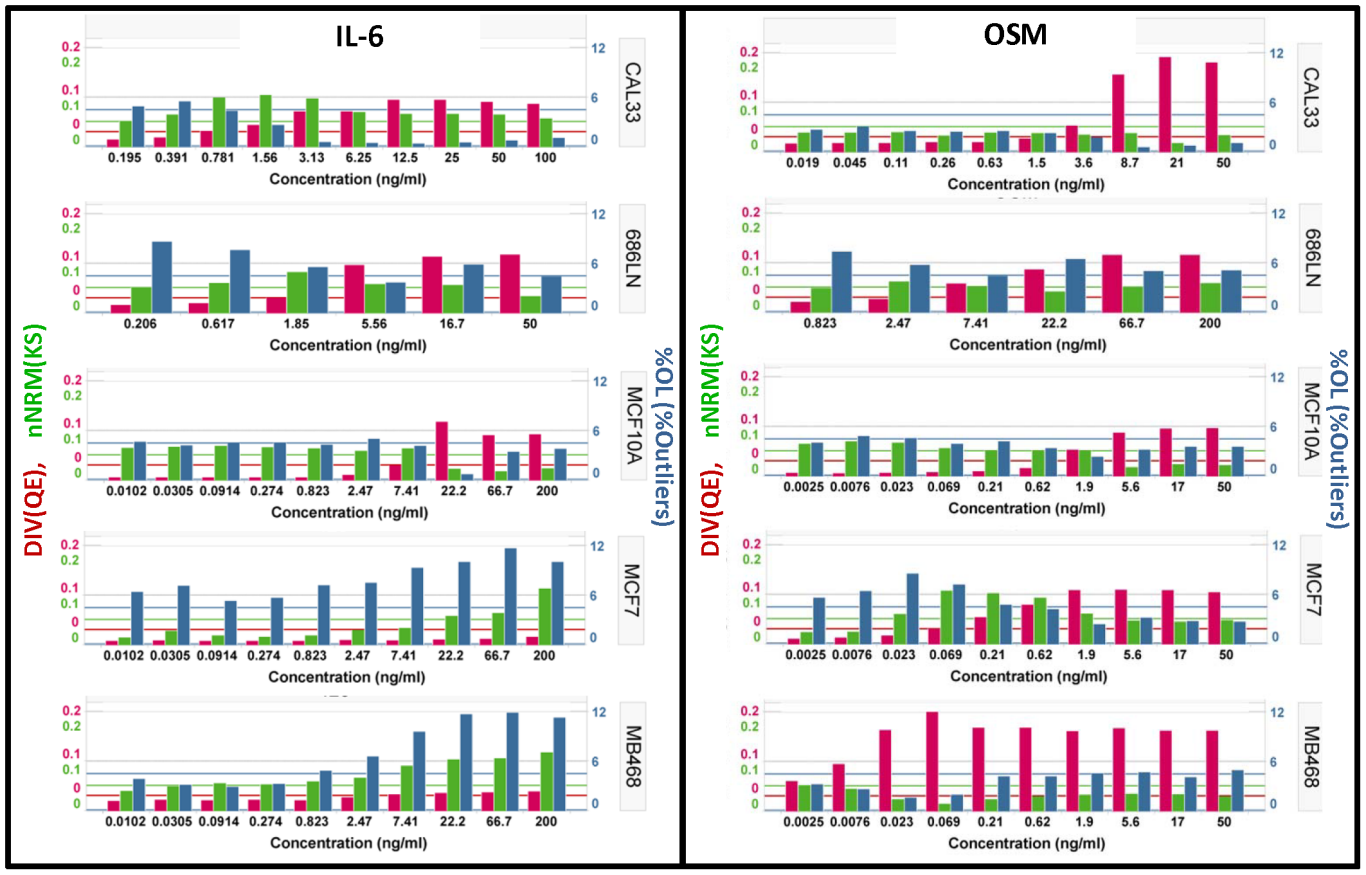
Comparison of the activation of STAT3 across 5 cell lines. Application of the HI's to the data in Figure 2. Left Panel) Activation of pSTAT3 by exposure to IL-6 for 15 min at the indicated concentrations. Right Panel) Activation of pSTAT3 by exposure to Oncostatin M for 15 min at the indicated concentrations. Red Bars) Diversity index (DIV) indicating the relative heterogeneity associated with the activation of pSTAT3. The horizontal red line indicates the selected threshold for classifying populations a heterogeneous. Green Bars) The non-Normality index (nNRM) indicating the extent of deviation from a single, normally distributed population. The green horizontal line indicates the selected threshold for classifying a population as having macro-heterogeneity. Blue Bars) The percent outliers (%OL) indicates the percentage of cells with an activity level that is above the upper inner fence or below the lower inner fence. The horizontal blue line indicates the selected threshold that is used to classify a population as having more than the expected number of outliers.

Based on the results described above we selected properties for characterization of the distributions that corresponded to the features that were identified as variable in the histo-box plots and descriptors that can be interpreted in a biologically meaningful way. Figure 4 defines the three selected descriptors of the distributions and the indices selected to quantify those features, cell diversity (DIV), non-normality (nNRM) and percent outliers (%OL). We compared the performance of several metrics for calculating DIV and nNRM, using model distributions and cell data, and found QE and KS gave the most consistent and robust results (Figure S7). The indices can be used for relative comparison or threshold values can be established for classification of samples. In this assay the Cal33 negative control wells (no IL-6) showed only a small percentage of cells with activated STAT3 while the majority were narrowly distributed (Figure 3). We used these ‘homogeneous’ negative control wells to establish a threshold value for DIV and nNRM, equal to the mean +3*SD of the well-to-well values of the index. For %OL the threshold was selected based on a normal distribution. The normal distribution has a UIF-LIF range of 4 SD (mean ± 2 SD) that contains 95.5% of the population and therefore the expected %OL is 4.5%. Therefore the threshold of >4.5% indicates more outliers than would be expected if the distribution were normal. Figure 5 shows the application of the selected threshold values for classification of wells as heterogeneous. To examine the suitability of the candidate heterogeneity indices and thresholds we used the data sets for IL-6 and OSM induction in the five cell lines (Figure 2). The results are presented as bar graphs of the three parameters (Figure 6). When stimulated with IL-6 (left panel), Cal33 cells exhibit a gradual increase in DIV, a consistently high nNRM and a decrease in %OL. On the other hand, OSM (right panel) has little effect on the HI's below 8.6 ng/ml but induces a nearly 2-fold greater increase in DIV, with essentially no change in nNRM or %OL, consistent with the distributions in Figure 3. The other cell lines exhibit different patterns of response to IL-6 and OSM. 686LN and MCF-10A cells respond essentially the same to IL-6 and OSM. MCF-7 and MDA-MB-468 cells respond to IL-6 with an increase in nNRM and %OL, but no increase in DIV, while OSM induces a significant increase in DIV, with a small increase in nNRM in the MCF-7 cells. It is interesting that the pattern of heterogeneity induced by OSM in MCF-7 cells is very similar to that induced by IL-6 in Cal33 cells. In nearly all cases OSM induces a more normally distributed response, which is still heterogeneous, while the response to IL-6 is much more variable.

The interpretation of the three HI's is accomplished by applying a binary decision tree (Figure 5) that classifies a population distribution as “homogeneous”, “homogeneous with outliers”, micro-heterogeneity, micro-heterogeneity with outliers, macro-heterogeneity, or macro-heterogeneity with outliers. We use “homogeneous” as a relative term, since cell populations always exhibit some heterogeneity.

**Figure 7:**
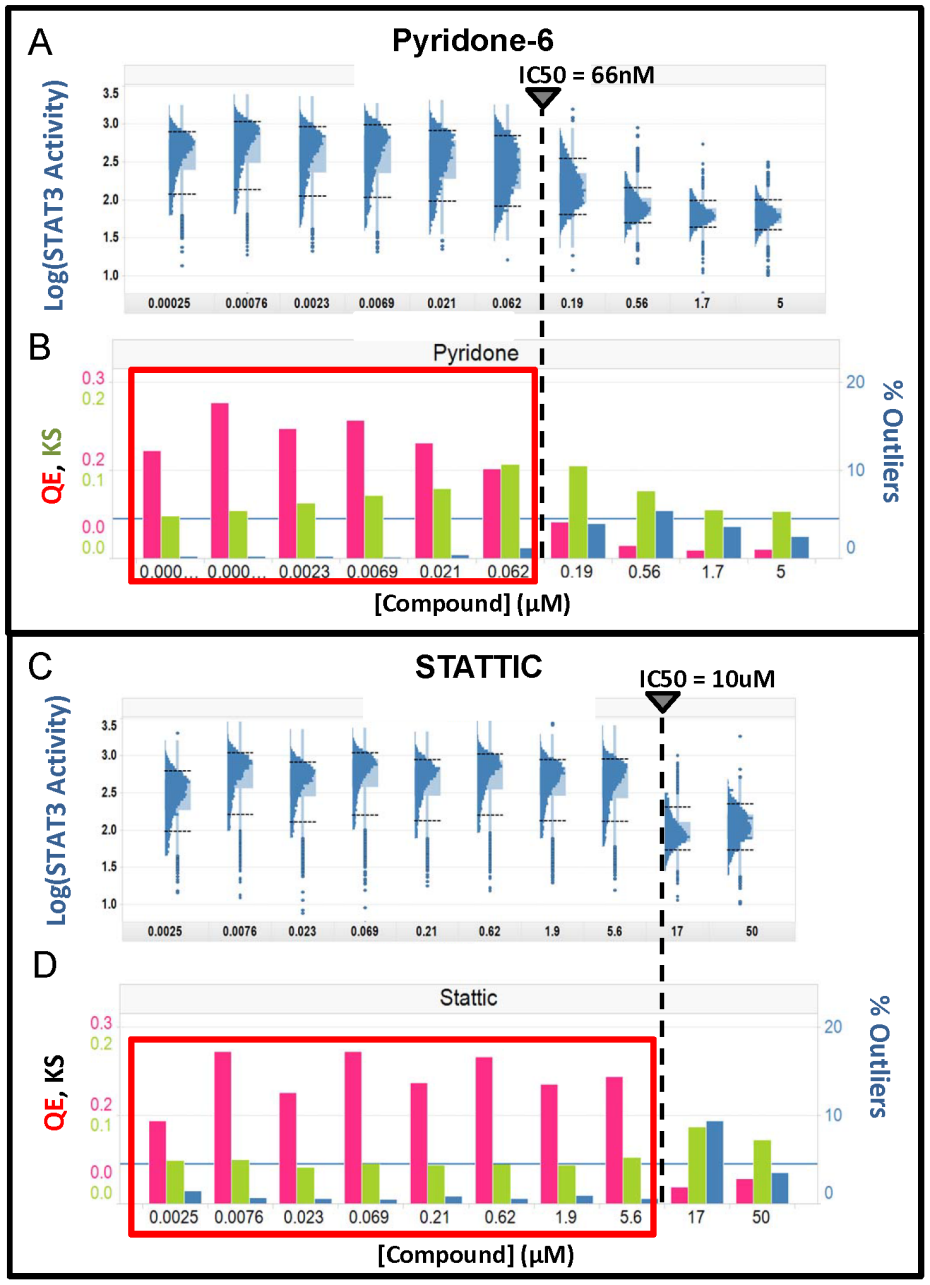
Heterogeneity in the response to inhibitors of STAT3 activation. Cal33 cells were exposed to Pyridone-6 (A&B) or STATTIC (C&D) at the indicated concentrations for 3 hours prior to stimulation with 50 ng/ml of IL-6. A) Inhibition by Pyridone-6. Log scaled distributions are plotted to normalize CV. B) Three heterogeneity parameters were calculated from the linear scaled data, DIV (red), nNRM (green) and %OL (blue). C) Log scaled distributions of inhibition by Stattic. D) The same three heterogeneity parameters are plotted for the linear scaled distributions of Stattic inhibition. The vertical dashed lines indicate the IC50 for the compounds calculated from the well-averaged signal intensities.

We evaluated the effect of two known inhibitors of STAT3 activation on the IL-6 stimulated distributions in Cal33 cells. Pyridone-6 is a pan-Janus-activated-kinase (Jak) inhibitor [51] and Stattic is reported to interact with the SH2 domain of STAT3, inhibiting dimerization and nuclear translocation [52]. Both compounds inhibited IL-6 induced STAT3 activation with IC50s of 0.066 µM and 10 µM, respectively (Figure 7 and Figure S8, DataArchive S2). Figure 7A and 7C display log-scaled histo-box plots of pyridone-6 and Stattic inhibition of Cal33 cells, respectively. The IC50s are shown as dashed lines. Pyridone-6 treated samples appear to have an increasing fraction of inhibited cells starting at the lowest concentration and a stable population of STAT3 activated cells up to about 0.1 µM, resulting in a broadening of the distribution. These trends are reflected in the HI's (Figure 7B). For pyridone-6 the DIV index is above threshold up to the IC50, but the increase in the nNRM index indicates the presence of differentially responding sub-populations of cells, macro-heterogeneity with outliers. Above 1 µM the cells are essentially all inhibited, except for a few outliers, some of which appear to be STAT3 activated cells.

Stattic, in contrast, displayed a stable population distribution with no evidence of inhibition until the concentration reaches the IC50 at which point there is a very steep inhibitory effect. Stattic showed essentially no change in HI's up to the IC50 indicating that nearly all the cells have essentially the same sensitivity to this compound, and therefore respond at the same dose level (Figure 7D). Thus, while pyridone-6 has a more potent IC50, Stattic has a more uniform effect as a modulator of STAT3 activation. In both cases the compounds show an increase in %OL above the IC50, exceeding the selected threshold of 4.5%, and persisting even at the highest dose tested. This indicates that neither compound is entirely effective at inhibiting the activation of STAT3.

An important consideration in the application of the HI's is the sample size requirements. To address this we performed power analysis for the HI's (Table S2). For the DIV and nNRM indices in this assay, to achieve a power level of 0.8 requires ≈900 and ≈1100 cells respectively. This number of cells is easily achievable in standard assay formats such as 384 well microplates. In this assay, which was implemented in the 384 well format, 1000 cells/well represents about 4 fields/well at 10x.

## Discussion

### The significance of heterogeneity in the development of therapeutics and diagnostics

Heterogeneity is a characteristic of cellular populations that is fundamental to biological processes including development, differentiation, and immune-mediated responses [9]. Analysis of heterogeneity is expected to be useful in a wide range of biological applications including the differentiation of stem cells and the development of assays in differentiated neuronal cells, where we would expect to find significant heterogeneity. Certainly, in the context of this cancer example, heterogeneity in the response to a potential therapy is “bad”, however, in other applications heterogeneity analysis may be essential to characterizing the response of a subpopulation of interest, or even as a primary readout for screening. When heterogeneity is associated with dysregulated genetic-based and/or non-genetic-based functions, it can play a critical role in the progression of complex diseases such as cancer [53], where intra-tumor heterogeneity poses a formidable challenge to the development of therapeutics [15], [54] as well as diagnostics [15], [25], [33]. Thus identifying, quantifying and characterizing heterogeneity in patient samples and disease relevant models for drug discovery using validated cell-by-cell analysis methods [15], [33], [53], [55] represents an important unmet need. To address this need we have defined and developed heterogeneity indices (HI's) (Figure 4) that enable the full potential of HCA and other cell-by-cell analytical methods. As a specific example, we applied these indices to identify, quantify, and characterize intrinsic heterogeneity in the activation of STAT3 in response to two cytokines and small molecule perturbagens. Based upon these results, we recommend a new paradigm for the application of these or similar HI's to the discovery of small molecule probes and therapeutics. Heterogeneity in the response to such probes may have important implications for understanding fundamental mechanisms of biological regulation and, as a mainstay in personalized medicine, lead to the development of novel therapeutic strategies for complex diseases (see below).

High Content Screening (HCS) was developed as a tool to automatically acquire, process, store, analyze and view large amounts of cellular data, creating an efficient platform for cell-by-cell analysis [22], [56]–[59]. However, the traditional focus in drug discovery on high throughput screening encouraged most researchers to focus on well average assays as a standard. This approach increased the throughput of screening but sacrificed the information on heterogeneity in the population [30]. As a result, although heterogeneity is widely recognized as a fundamental characteristic of biological systems, relatively little is known about the nature of heterogeneity in the cellular or tissue response to current pharmaceuticals.

Although a well average assay may exhibit a very good Z’, and therefore a high degree of reproducibility [27], [28], the cell-to-cell heterogeneity within a well can be significant (Fig 1, 2, 3, 6, 7). In developing new drugs it is not sufficient to modulate the “average” cell if heterogeneity exists, particularly for cancer therapeutics. Thus we aimed to identify a simple set of metrics, the HI's, that could be automatically calculated and reported along with the standard well-level read-outs of mean and SD, and the well-to-well, plate-to-plate and day-to-day metrics of Z’, S/B, and CV, to rapidly determine if heterogeneity exists and to quantify the extent of the heterogeneity (Figures 4, 5, 6, 7).

As HCA is utilized more extensively to quantitate cellular heterogeneity, there must be a focus on the development of quality control standards and practices such as those that have been successfully implemented in flow cytometry. To distinguish the “system” variability (includes sample preparation, instrument response and algorithm performance) from the variability in biological responses (i.e. intrinsic biological heterogeneity) we used fluorescent calibration beads with a narrow and well characterized distribution (Figure S3 and Table S1). These highly uniform beads established that the minimum limit of variability in measurements of intensity in this assay, CV of 5–8%, was well below the observed heterogeneity in STAT3 activation. Minimizing the “system” variability is critical to performing quantitative fluorescence imaging and has been discussed in detail [60]–[62].

### Selecting heterogeneity indices (HI's) to apply to single cell analyses

We considered three properties of the distribution of data that are significant in the biological interpretation of heterogeneity and selected HI's to describe each: 1) How variable is the response? 2) Is there more than one type of response? and 3) Are there outlier cells that respond differently? We evaluated several statistical measures of distribution width (diversity) including the IQR, Shannon Entropy (SE) [63], Differential Entropy(DE) and Quadratic Entropy (QE) (see Figure S7). We used the QE, which has been shown to provide a quantitative measure of species diversity and incorporates information not only on the number of different species in a population, but also on the magnitude of the differences between biological species [64], [65]. The QE has also been shown to be useful in quantitation of the diversity of cellular phenotypes in cancer tissue sections for diagnostic application [33], and we have extended that use to the characterization of cellular diversity (DIV) in HCA assays.

To further characterize the population responses with respect to the presence of subpopulations (i.e., discrete phenotypic cell states) we adopted the definition of macro- and micro-heterogeneity proposed by Huang [9]. Macro-heterogeneity refers to the variability in one or more cell traits that results in discrete phenotypes and a multimodal distribution. Examples of macro-heterogeneity include the distinct states of progenitor vs. differentiated cells, the phases of the cell cycle and the time dependent changes in the intracellular distribution of proteins such as transcription factors. Micro-heterogeneity is defined as the apparently continuous random variation in a single phenotype, leading to a normal (or log-normal) distribution of the cell trait. Examples include population noise, such as the prolonged expression level of a protein during development, and temporal noise based on stochastic fluctuations of a cell trait within a single cell over time that are not usually synchronized between cells in the population.[9]. Based on these definitions, the distinction between micro- and macro-heterogeneity is equivalent to a normality test. We evaluated several potential measures of distribution shape including skewness, kurtosis, mean-median ratio and the Kolmogorov-Smirnov (KS) test relative to a normal distribution with the same mean and SD, also known as the Lilliefors test [66] (see Figure S7). The KS test is an established measure of normality [67], [68] and the use of KS analysis is well known in HCA [30], [35], [36], [69]–[71]. The deviation of the distribution from micro- to macro-heterogeneity, results in an increase in the KS statistic, which we use as a non-normality index (nNRM), indicating that there may be more than one mechanism of response or that the cells may be in more than one state and therefore should be further evaluated.

The third index of heterogeneity quantifies the percentage of outliers. The presence of outlier cells that respond distinctly from the majority is usually completely ignored in HCA. These outliers may be critically important in the development of therapeutics, especially in cancer, where a small number of resistant sub-clones may exist prior to treatment, then undergo positive selection, resulting in only a transient beneficial response and consequently result in high rates of relapse [72]. The percent outlier index (%OL) was chosen based on the standard statistical definition of outliers used in box plots: samples outside the range from the lower inner fence to the upper inner fence. Other choices of outlier definitions could also be applied, but this particular definition is consistent with our choice of the histo-box plot for reviewing heterogeneity. The biological interpretation of outliers is challenging due to the relatively small numbers, but requires further evaluation when detected.

The combination of these three heterogeneity indices (HI's) can be used to classify the heterogeneity in a cell population using a binary decision tree as shown in Figure 5. The criteria for selecting classification threshold values will vary depending on the project. For example, in the IL-6 activation assay, the negative control wells were nearly ‘homogeneous’ while in the inhibition assay, where all wells contained IL-6, the maximally inhibited wells were most nearly ‘homogeneous’. We chose to use threshold values that were 3 SD above the mean DIV or nNRM in replicate control wells as indicating a substantial increase in heterogeneity relative to the control. Alternatively, for the nNRM index an absolute threshold could be defined based on the critical values for the KS test [66], [68]. To achieve 99% confidence in the determination of non-normality, the KS statistic must be ≥1.031/√N, where N is the sample size. In this study the minimum sample size was about 2,000 cells per well which results in a critical KS value of 0.02. We used a more conservative threshold of 0.05. For %OL we defined an absolute threshold based on the percentage of a normally distributed population that would be classified as outliers (4.5%). For screening or other large scale profiling applications, these indices can be sorted, clustered or viewed as heat maps to identify cell population profiles that indicate more complex biology. For visual analysis of the distribution we found the histo-box plot to be much more useful than the standard box plot. For multiparameter assays, the heterogeneity indices can be evaluated on each readout, or a dimension reduction approach such as principal component analysis [73], [74] can be applied first and the HI's calculated for the principal components.

### Application and impact of heterogeneity analysis in drug discovery

**Figure 8:**
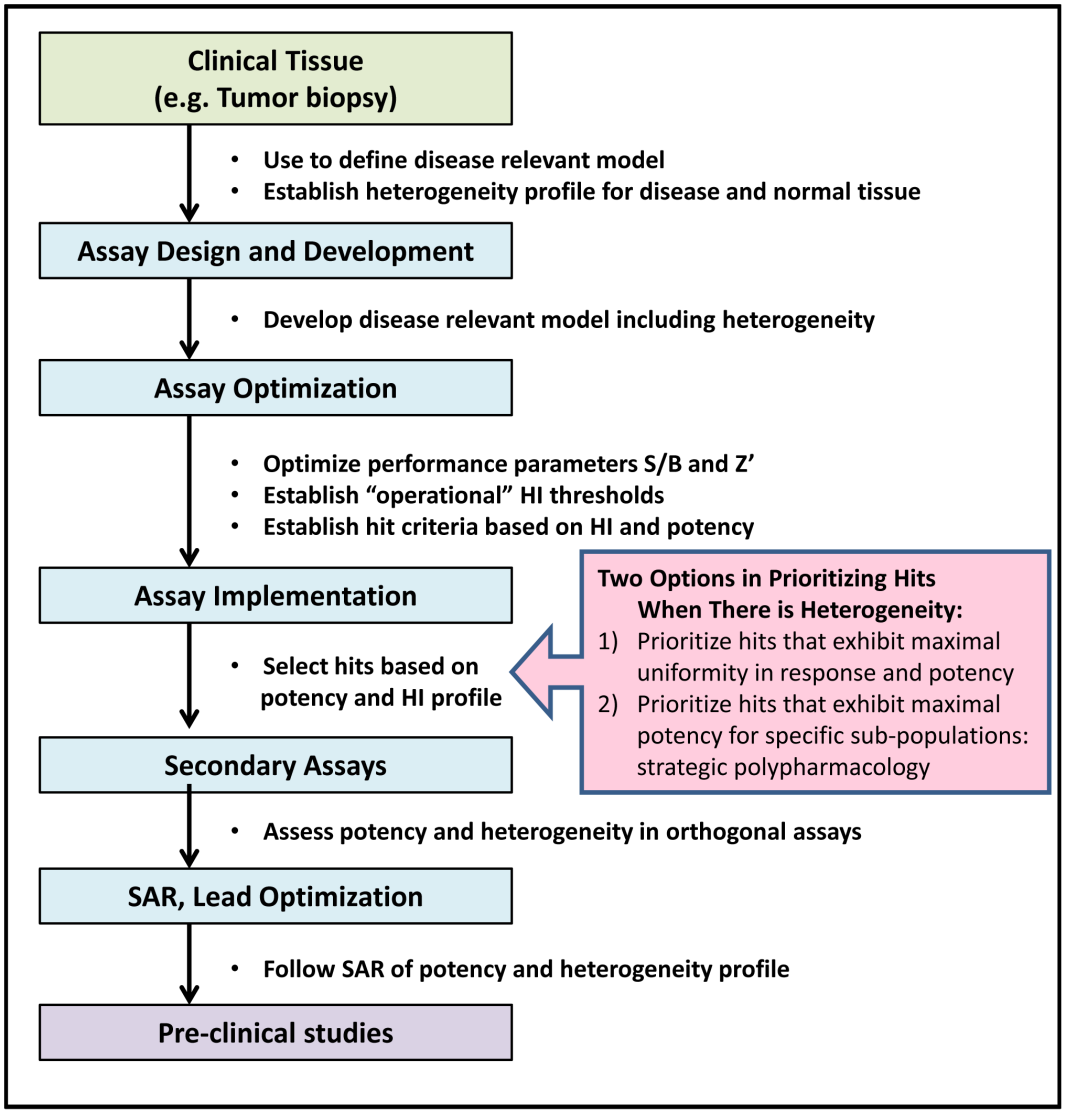
Heterogeneity analysis applied throughout the early drug discovery process. Heterogeneity analysis is required to guide decisions throughout the drug discovery process, beginning with defining disease relevant biology in clinical samples, and establishing benchmarks for subsequent analyses. Next disease relevant models, which by necessity will be heterogeneous, are developed and optimized. Heterogeneity is characterized in the models, and thresholds for HI's are established along with potency criteria to select hits. Screening hits are advanced to secondary assays based on their potency and HI profile. Heterogeneity of response to compounds will be model dependent, and assessing heterogeneity in orthogonal secondary assays will provide insights into understanding the MOA. Monitoring the heterogeneity profile during SAR and lead optimization is essential to keeping lead development on target and mechanism of the disease relevant biology.

For the reasons stated above it is important to apply heterogeneity analysis throughout the early drug discovery process from assay design and implementation through secondary screens, SAR analysis and into pre-clinical studies (Figure 8). The development of disease relevant models and assays begins with the analysis of patient samples to identify suitable biomarkers and assay readouts, and to establish differences in the organization and heterogeneity profiles of those biomarkers in diseased and normal tissues. Physiologically relevant models that recapitulate the disease state may require more complex architecture, including multiple cell types, which also lead to heterogeneity in assay readout(s). The methods proposed here can be applied in both cases to characterize and track heterogeneity, and to optimize the model. For example, in the Cal33 assay used here, not all of the cells responded to IL-6 activation of STAT3, whereas all cells responded to OSM (Figure 3). Choosing IL-6 stimulation for lead identification may limit the screen to selecting compounds that have mechanisms present in only a subset of cells (i.e., those that are IL-6 dependent), ultimately reducing therapeutic efficacy and necessitating combination strategies (see below). A more appropriate assay may be one using OSM and/or a combination of cytokines as the inducer, but the choice should optimally be driven by an understanding of the pathway and the role heterogeneity plays in the dysregulation of STAT3 in cancer tissue.

During implementation of an assay in a screening campaign, HI's would be reported alongside the compound potency and assay performance statistics (Figure 8 and S9), flagging compound concentrations that exceed the established thresholds (Figure 5). A DIV that indicates heterogeneity will be an alert that a compound induced variable responses within the cell population, and the nNRM and %OL will further classify the heterogeneity. Applying the decision tree described in Figure 5 to the pyridone-6 data in Figures 7 and S9, the DIV index indicates heterogeneity below 0.56 µM, while the nNRM index shows a concentration dependent macro-heterogeneity in the inhibitory response (nNRM HI>0.05) indicating sub-populations of cells with different sensitivities to pyridone-6 inhibition. Furthermore, both compounds show an increase in %OL above the IC50, indicating that some cells continue to activate STAT3, even in the presence of inhibitor. Although representing only a small percentage of the cells, resistance to treatment may have important implications in cancer therapy. The %OL feature should provide additional information for selection and optimization of hits and leads, as well as a readout that could be used to screen combinations of compounds for improved efficacy with respect to outlier cells.

In drug development, compounds where macro-heterogeneity is identified would need to be further studied, perhaps starting with the histo-box plot. Compounds exhibiting heterogeneity present two options: (1) deprioritize in favor of compounds that modulate the cell population more uniformly; or (2) select the compounds for efficacy in a specific sub-population for use in a combination therapy strategy. In this study pyridone-6 and Stattic exhibited very different, dose dependent heterogeneity profiles, consistent with their different reported mechanisms of action. The objective of monitoring heterogeneity in secondary assays should be to identify potential differences in MOA between lead compounds and to more fully characterize the range of cellular responses, enabling more informed decisions in selecting compounds to advance through drug development.

Finally, it is important to follow the heterogeneity profile while investigating SAR in the lead optimization stage to ensure that changes in the compound structure do not introduce additional or undesirable heterogeneity in the response, implying additional mechanisms of action. Furthermore, the heterogeneity profile can be used in combination with biological potency to drive the SAR of a lead series towards a non-disease profile.

### Importance of heterogeneity analysis in understanding biological regulation and in developing optimized cancer therapeutic strategies

Darwinian-like clonal evolution significantly contributes to the genetic heterogeneity within tumors, which contributes to the observed phenotypic diversity [53], [75], [76]. Additional intra-tumoral phenotypic diversity results from epigenetic changes [43], [53], [75] or as a consequence of heterotypic signaling within an abnormal micro-environment [76], [77]. This phenotypic diversity and plasticity, in conjunction with the complexity of STAT3 signaling and regulation that involves crosstalk with several other pathways (e.g. PI3K, RAS, NFκB, NOTCH) [78]–[83], enables small molecule perturbagens to induce both micro- and macro-heterogeneous responses. For example, fluctuations in the expression of signaling components can alter the kinetics of a specific step targeted by a small molecule, inducing micro-heterogeneity. Alternatively macro-heterogeneity, such as evidenced by the presence of apparently non-responder subpopulations, could result from changes in protein expression that result in dysregulation of the crosstalk involved in negative feedback or the activation of compensatory pathways. In fact these latter two mechanisms are among the most common for generating resistance to targeted therapies in cancer [72], [84]–[87]. Thus, heterogeneity presents a major challenge to optimizing therapeutic regimens, as the targeting of a predominant tumor subpopulation often only provides transient benefit and will inevitably result in the emergence of resistant populations, and relapse [72].

Recent studies suggest that knowledge of the tumor composition and the response of component subpopulations to single drugs, in conjunction with computational and experimental modeling, can identify drug combinations that minimize the outgrowth of resistant subpopulations in tumors, while enhancing tumor free survival in mice [8], [88]. Importantly, the experimentally validated computational simulations demonstrated that the optimal drug combination predicted depended on whether the entire tumor population, or only a particular subpopulation, was examined. These results further emphasize the need to incorporate intra-tumor heterogeneity and the expected evolutionary trajectories into rational drug combination design.

The development of comprehensive, unbiased target based mutagenesis and genome-wide gain- and loss-of-function technologies that can anticipate clinically relevant resistance, represents an alternative, or perhaps complementary approach to modeling and therapeutically addressing tumor heterogeneity [72], [90], [91]. It is likely that the future of personalized cancer medicine will involve a comprehensive bioinformatics analysis of a biopsy that will reveal the presence of distinct cell populations [6], [89] and consultation of an established drug-genotype database that will allow clinicians to computationally determine optimal, patient-specific combination therapies. We hypothesize that heterogeneity analysis will be essential to the implementation of a QSP-driven approach that includes the highly coordinated, parallel optimization of complementary lead structures, where each structure has a clinically relevant resistance profile that is addressed by its counterpart, which will lead to polypharmacologic therapies that effectively drive the tumor into a state of ‘checkmate’, thereby providing sustainable remissions and higher cure rates.

## Supporting Information

Figure S1 **Time course and dose-response of the activation of STAT3 in Cal33 cells.** A) Dose-response of IL-6 indicated by logistic regression curve fit to well average intensities in 3 replicate wells. Error bars (±1σ of the cell-by-cell intensities) indicate the high variability in the cell-to-cell STAT3 activity B) Dose-response of OSM activation of STAT3 fit as in A. Error bars (±1σ of the cellular intensities) again indicate a high degree of heterogeneity. C) Well average STAT3 activity in Cal33 cells that were exposed to the indicated concentrations of IL-6 for the times indicated by color. Error bars (±1σ of the 3 replicate wells) indicate the assay is highly reproducible despite cellular heterogeneity. D) Same as C) except cells were exposed to the indicated concentrations of OSM. Time indicated by colors.
(TIF)

Figure S2 **Use of Histo-Box plot for visualization of distributions.** A) Histograms of simulated, normally distributed data with the indicated mean and a CV = 30% for all unimodal distributions. The 2 bimodal distributions combine the distributions with means of 500 and 10 and CV = 30%, equally weighted (‘500+10’) and with a 90∶10 split (‘500(90%)+10(10%)’). Each distribution consists of 2000 random numbers. B) The same distributions as in A, logarithmically scaled. The key defines the reference points labeled on the plot (A).
(TIF)

Figure S3 **Quantitating the Reproducibility of Intensity Measurements by Flow and Imaging Cytometry.** A) Histogram of the total intensity of 2 µm flow cytometry standard beads measured by flow cytometry shows peaks for single beads (red, CV = 2.8) and double beads (blue). B) Histogram of the same beads centrifuged in the wells of a 384 well microplate and imaged to measure total bead intensity also shows peaks for single beads (red, CV = 5.2) and double beads (blue). C) The histogram of total nuclear intensity in Cal33 cells fixed in suspension, labeled with Hoechst and measured by flow cytometry. Cell cycle modeling identifies 3 subpopulations, G0/G1 (red), S (hashed), and G2/M (blue). D) The histogram and cell cycle modeling of the same cells centrifuged in the wells of a 384 well microplate then imaged and analyzed for total nuclear intensity.
(TIF)

Figure S4 **The distributions of STAT3 activation for cell cycle subpopulations.** Histograms of STAT3 activation in unstimulated and IL-6 stimulated Cal33 cells (the cell cycle states identified in the legend). Inset DNA histogram of the cumulative population shows the mapping of DNA intensities to cell cycle states.
(TIF)

Figure S5 **Western Blot Analysis of Receptor Expression.** A) Western blots of receptor expression in five cell lines, with Tubulin as a control. B) Quantitation of total density in western blot bands for the IL-6 receptor, relative to Cal33 cells. C) Quantitation of OSM receptor expression, relative to Cal33. D) Quantitation of IFNγ receptor expression, relative to Cal33.
(TIF)

Figure S6 **STAT3 activation by Combinations of Cytokines.** To assess the interaction between the IL-6 and OSM pathways, Cal33 cells were exposed to combinations of IL-6 and OSM for 15 min. Top Row) The activation of STAT3 by IL-6 alone. Left Column) The activation of STAT3 by OSM alone. The arrows point to the location of the population non-responsive to IL-6. Note that with the addition of OSM all cells treated with IL-6 show activated STAT3.
(TIF)

Figure S7 **Evaluation of Potential indices of Diversity and Normality.** A) Model distributions and cell data were used to evaluate the performance of selected metrics for characterizing the distributions. The 50∶50 mix consists of 2 unit normal distributions of equal population that are separated by ‘d’ standard deviations (sd). The 90∶10 mix consists of 2 unit normal distributions with 90% and 10% of the population, separated by ‘d’ standard deviations. B) Selected Diversity and Normality indices were used to evaluate the model distributions for values of ‘d’ ranging from 0–10 sd, and the Cal33 data for IL-6 and OSM stimulation. For the Cal33 data, the diversity indices all show similar response, while the model data show some key differences. The IQR (interquartile range) is not sensitive to the small 10% subpopulation, and the SE (Shannon entropy) and DE (Differential entropy, the Shannon entropy for a continuous distribution function) both plateau when the 2 populations separate. Only the QE (Quadratic entropy) shows a steady increase for both distributions. Again, the general pattern of the ‘Normality’ measures is similar for the Cal33 data, but the model data show key differences. The skewness and MMR (mean/median) are insensitive to the 50∶50 population because it is symmetric, The kurtosis and KS statistic are sensitive to the variation in both distributions, however the KS was preferred due to its direct interpretation as a measure of normality.
(TIF)

Figure S8 **Dose dependence of inhibitors of STAT3 activation by IL-6.** STAT3 activation in Cal33 cells is inhibited by Pyridone-6 (•) with an IC50 = 66 nM or STATTIC (▪) with an IC50  = 10 µM.
(TIF)

Figure S9 **Integrating heterogeneity analysis into phenotypic screening.** Heterogeneity indices are evaluated during assay development and thresholds determined based on the goals of the project. For drug discovery and cell/tissue profiling programs that encounter phenotypic heterogeneity, HCS images are analyzed to generate the features, statistical parameters and HI's. For samples or treatments with low Diversity (DIV) or a normal distribution (low nNRM) standard statistics can be used. A well or sample with a high HI and high nNRM or high %OL would require more detailed analysis of the heterogeneity. If the observed heterogeneity is biologically important in the context of the project, further experiments aimed at understanding its mechanism may lead to discovery of new targets or diagnostic biomarkers. For pyridone-6, the DIV index for concentrations below 5 µM indicates a high degree of heterogeneity (HI>0.03 from Figure 5) which is further characterized as macro-heterogeneity since the nNRM indices are >0.5. At 5 µM the DIV indicates a homogeneous population with low heterogeneity. In all cases the %OL is below the HI threshold in Figure 5. The high heterogeneity indices suggest further studies are needed to understand the activity of pyridone-6 on these cells.
(TIF)

DataArchive S1 **Data for the distribution of STAT3 activation in 5 cell lines stimulated with 10 concentrations of IL-6 or OSM as plotted in** **Figure 2** **.** Data provided as a ZIP archive.
(ZIP)

DataArchive S2 **Data for the inhibition of IL-6 induced STAT3 activation by Pyridone-6 and Stattic as plotted in** **Figure 7** **.** Data provided as a ZIP archive.
(ZIP)

Table S1 **Reproducibility of Intensity Measures.** Flow cytometry standard beads and Cal33 cells were used to quantify the reproducibility of imaging intensity measurements on cells and cell sized objects. Samples of beads or cells were split and run on either the ArrayScan HCA system or a flow cytometer for reference. For beads, Ratio is (mean doublet total intensity)/(mean singlet total intensity). For Cal33 cells, Ratio is (mean G2/M total nuclear intensity)/(mean G0/G1 total nuclear intensity).
(DOCX)

Table S2 **Power analysis of HI measures.** Replicate measures on 3 different days were used to determine the number of cells required to achieve a power of 0.8 for the CV, KS and QE measures of the distributions of STAT3 activity in Cal33 cells.
(DOCX)

## References

[1] Li RX, Zeng R (2009) Dynamic proteomics for investigating the response of individual cancer cells under drug action. Expert Rev Proteomics 6: 19–21. 19210123 10.1586/14789450.6.1.19

[2] Greaves M, Maley CC (2012) Clonal evolution in cancer. Nature 481: 306–313. 22258609 10.1038/nature10762 PMC3367003

[3] Almendro V, Marusyk A, Polyak K (2013) Cellular Heterogeneity and Molecular Evolution in Cancer. Annual Review of Pathology: Mechanisms of Disease 8: 277–302. 10.1146/annurev-pathol-020712-163923 23092187

[4] Meacham CE, Morrison SJ (2013) Tumour heterogeneity and cancer cell plasticity. Nature 501: 328–337. 24048065 10.1038/nature12624 PMC4521623

[5] Junttila MR, de Sauvage FJ (2013) Influence of tumour micro-environment heterogeneity on therapeutic response. Nature 501: 346–354. 24048067 10.1038/nature12626

[6] Fedele C, Tothill RW, McArthur GA (2014) Navigating the challenge of tumor heterogeneity in cancer therapy. Cancer Discov 4: 146–148. 24501303 10.1158/2159-8290.CD-13-1042

[7] Fang C, Avis I, Salomon D, Cuttitta F (2013) Novel Phenotypic Fluorescent Three-Dimensional Platforms for High-throughput Drug Screening and Personalized Chemotherapy. J Cancer 4: 402–415. 23833685 10.7150/jca.6780 PMC3701810

[8] Zhao B, Pritchard JR, Lauffenburger DA, Hemann MT (2014) Addressing Genetic Tumor Heterogeneity through Computationally Predictive Combination Therapy. Cancer Discovery 4: 166–174. 24318931 10.1158/2159-8290.CD-13-0465 PMC3975231

[9] Huang S (2009) Non-genetic heterogeneity of cells in development: more than just noise. Development 136: 3853–3862. 19906852 10.1242/dev.035139 PMC2778736

[10] Rubin H (1990) The significance of biological heterogeneity. Cancer Metastasis Rev 9: 1–20. 2208565 10.1007/BF00047585

[11] Bright GR, Whitaker JE, Haugland RP, Taylor DL (1989) Heterogeneity of the changes in cytoplasmic pH upon serum stimulation of quiescent fibroblasts. J Cell Physiol 141: 410–419. 2478571 10.1002/jcp.1041410223

[12] Burrell RA, McGranahan N, Bartek J, Swanton C (2013) The causes and consequences of genetic heterogeneity in cancer evolution. Nature 501: 338–345. 24048066 10.1038/nature12625

[13] Turner NC, Reis-Filho JS (2012) Genetic heterogeneity and cancer drug resistance. The Lancet Oncology 13: e178–e185. 22469128 10.1016/S1470-2045(11)70335-7

[14] Brock A, Chang H, Huang S (2009) Non-genetic heterogeneity—a mutation-independent driving force for the somatic evolution of tumours. Nat Rev Genet 10: 336–342. 19337290 10.1038/nrg2556

[15] Gough A, Lezon T, Faeder JR, Chennubhotla C, Murphy RF, et al. (2014) High Content Analysis with Cellular and Tissue Systems Biology: A Bridge Between Cancer Cell Biology and Tissue-Based Diagnostics. In: Gray J, editor. The Molecular Basis of Cancer. 4 ed. Philadelphia, PA: Elsevier.

[16] Niepel M, Spencer SL, Sorger PK (2009) Non-genetic cell-to-cell variability and the consequences for pharmacology. Curr Opin Chem Biol 13: 556–561. 19833543 10.1016/j.cbpa.2009.09.015 PMC2975492

[17] Cohen AA, Geva-Zatorsky N, Eden E, Frenkel-Morgenstern M, Issaeva I, et al (2008) Dynamic proteomics of individual cancer cells in response to a drug. Science 322: 1511–1516. 19023046 10.1126/science.1160165

[18] Barabasi AL, Gulbahce N, Loscalzo J (2011) Network medicine: a network-based approach to human disease. Nat Rev Genet 12: 56–68. 21164525 10.1038/nrg2918 PMC3140052

[19] Pujol A, Mosca R, Farres J, Aloy P (2010) Unveiling the role of network and systems biology in drug discovery. Trends Pharmacol Sci 31: 115–123. 20117850 10.1016/j.tips.2009.11.006

[20] Auffray C, Chen Z, Hood L (2009) Systems medicine: the future of medical genomics and healthcare. Genome medicine 1: 2. 19348689 10.1186/gm2 PMC2651587

[21] Taylor DL (2007) Past, present, and future of high content screening and the field of cellomics. Methods Mol Biol 356: 3–18. 16988391 10.1385/1-59745-217-3:3

[22] Thomas N (2010) High-content screening: a decade of evolution. J Biomol Screening 15: 1–7. 10.1177/1087057109353790 20008124

[23] Bodenmiller B, Zunder ER, Finck R, Chen TJ, Savig ES, et al (2012) Multiplexed mass cytometry profiling of cellular states perturbed by small-molecule regulators. Nat Biotechnol 30: 858–867. 22902532 10.1038/nbt.2317 PMC3627543

[24] Wang D, Bodovitz S (2010) Single cell analysis: the new frontier in ‘omics’. Trends Biotechnol 28: 281–290. 20434785 10.1016/j.tibtech.2010.03.002 PMC2876223

[25] Critchley-Thorne RJ, Miller SM, Taylor DL, Lingle WL (2009) Applications of Cellular Systems Biology in Breast Cancer Patient Stratification and Diagnostics. Combinatorial Chemistry and High Throughput Screening 12: 860–869. 19531004 10.2174/138620709789383222

[26] Bray MA, Carpenter A (2012) Advanced Assay Development Guidelines for Image-Based High Content Screening and Analysis. In: Sittampalam GS, Gal-Edd N, Arkin M, Auld D, Austin C et al., editors. Assay Guidance Manual. Bethesda (MD): Eli Lilly & Company and the National Center for Advancing Translational Sciences [Available from: http://www.ncbi.nlm.nih.gov/books/NBK126174/]. 23469374

[27] Zhang J-H, Chung TDY, Oldenburg KR (1999) A Simple Statistical Parameter for Use in Evaluation and Validation of High Throughput Screening Assays. J Biomol Screen 4: 67–73. 10838414 10.1177/108705719900400206

[28] Zhang XD (2007) A pair of new statistical parameters for quality control in RNA interference high-throughput screening assays. Genomics 89: 552–561. 17276655 10.1016/j.ygeno.2006.12.014

[29] Azegrouz H, Karemore G, Torres A, Alaiz CM, Gonzalez AM, et al. (2013) Cell-Based Fuzzy Metrics Enhance High-Content Screening (HCS) Assay Robustness. J Biomol Screen. 10.1177/1087057113501554 24045580

[30] Haney SA (2014) Rapid Assessment and Visualization of Normality in High-Content and Other Cell-Level Data and Its Impact on the Interpretation of Experimental Results. J Biomol Screen. 10.1177/1087057114526432 24652972

[31] Loo LH, Lin HJ, Steininger RJ 3rd, Wang Y, Wu LF, et al (2009) An approach for extensibly profiling the molecular states of cellular subpopulations. Nat Methods 6: 759–765. 19767759 10.1038/nmeth.1375 PMC2818727

[32] Hasenauer J, Heinrich J, Doszczak M, Scheurich P, Weiskopf D, et al (2012) A visual analytics approach for models of heterogeneous cell populations. EURASIP J Bioinform Syst Biol 2012: 4. 22651376 10.1186/1687-4153-2012-4 PMC3403928

[33] Potts SJ, Krueger JS, Landis ND, Eberhard DA, Young GD, et al (2012) Evaluating tumor heterogeneity in immunohistochemistry-stained breast cancer tissue. Lab Invest 92: 1342–1357. 22801299 10.1038/labinvest.2012.91

[34] Boland MV, Murphy RF (2001) A neural network classifier capable of recognizing the patterns of all major subcellular structures in fluorescence microscope images of HeLa cells. Bioinformatics 17: 1213–1223. 11751230 10.1093/bioinformatics/17.12.1213

[35] Giuliano KA, Chen Y-T, Taylor DL (2004) High-Content Screening with siRNA Optimizes a Cell Biological Approach to Drug Discovery: Defining the Role of P53 Activation in the Cellular Response to Anticancer Drugs. J Biomol Screen 9: 557–568. 15475475 10.1177/1087057104265387

[36] Perlman ZE, Slack MD, Feng Y, Mitchison TJ, Wu LF, et al (2004) Multidimensional drug profiling by automated microscopy. Science 306: 1194–1198. 15539606 10.1126/science.1100709

[37] Giuliano KA, Cheung WS, Curran DP, Day BW, Kassick AJ, et al (2005) Systems Cell Biology Knowledge Created from High Content Screening. Assay Drug Dev Technol 3: 501–514. 16305307 10.1089/adt.2005.3.501

[38] Loo LH, Wu LF, Altschuler SJ (2007) Image-based multivariate profiling of drug responses from single cells. Nat Methods 4: 445–453. 17401369 10.1038/nmeth1032

[39] Gascoigne KE, Taylor SS (2008) Cancer cells display profound intra- and interline variation following prolonged exposure to antimitotic drugs. Cancer Cell 14: 111–122. 18656424 10.1016/j.ccr.2008.07.002

[40] Slack MD, Martinez ED, Wu LF, Altschuler SJ (2008) Characterizing heterogeneous cellular responses to perturbations. Proceedings of the National Academy of Sciences of the United States of America 105: 19306–19311. 19052231 10.1073/pnas.0807038105 PMC2614757

[41] Loo LH, Lin HJ, Singh DK, Lyons KM, Altschuler SJ, et al (2009) Heterogeneity in the physiological states and pharmacological responses of differentiating 3T3-L1 preadipocytes. J Cell Biol 187: 375–384. 19948481 10.1083/jcb.200904140 PMC2779244

[42] Singh DK, Ku CJ, Wichaidit C, Steininger RJ 3rd, Wu LF, et al (2010) Patterns of basal signaling heterogeneity can distinguish cellular populations with different drug sensitivities. Mol Syst Biol 6: 369. 20461076 10.1038/msb.2010.22 PMC2890326

[43] Gupta PB, Fillmore CM, Jiang G, Shapira SD, Tao K, et al (2011) Stochastic state transitions give rise to phenotypic equilibrium in populations of cancer cells. Cell 146: 633–644. 21854987 10.1016/j.cell.2011.07.026

[44] Gioanni J, Fischel JL, Lambert JC, Demard F, Mazeau C, et al (1988) Two new human tumor cell lines derived from squamous cell carcinomas of the tongue: establishment, characterization and response to cytotoxic treatment. European journal of cancer & clinical oncology 24: 1445–1455. 3181269 10.1016/0277-5379(88)90335-5

[45] Bauer VL, Hieber L, Schaeffner Q, Weber J, Braselmann H, et al (2010) Establishment and Molecular Cytogenetic Characterization of a Cell Culture Model of Head and Neck Squamous Cell Carcinoma (HNSCC). Genes 1: 388–412. 24710094 10.3390/genes1030388 PMC3966227

[46] Hintze JL, Nelson RD (1998) Violin plots: A box plot-density trace synergism. American Statistician 52: 181–184.

[47] Kampstra P (2008) Beanplot: A Boxplot Alternative for Visual Comparison of Distributions. Journal of Statistical Software, Code Snippets 28: 1–9.

[48] Team RC (2012) R: A language and environment for statistical computing. Vienna, Austria: R Foundation for Statistical Computing.

[49] Johnston PA, Sen M, Hua Y, Camarco D, Shun TY, et al. (2013) High-Content pSTAT3/1 Imaging Assays to Screen for Selective Inhibitors of STAT3 Pathway Activation in Head and Neck Cancer Cell Lines. Assay Drug Dev Technol. 10.1089/adt.2013.524 PMC3934522 24127660

[50] Limpert E, Stahel WA, Abbt M (2001) Log-normal Distributions across the Sciences: Keys and Clues. Bioscience 51: 341–352.

[51] Pedranzini L, Dechow T, Berishaj M, Comenzo R, Zhou P, et al (2006) Pyridone 6, a pan-Janus-activated kinase inhibitor, induces growth inhibition of multiple myeloma cells. Cancer Res 66: 9714–9721. 17018630 10.1158/0008-5472.CAN-05-4280

[52] Schust J, Sperl B, Hollis A, Mayer TU, Berg T (2006) Stattic: a small-molecule inhibitor of STAT3 activation and dimerization. Chem Biol 13: 1235–1242. 17114005 10.1016/j.chembiol.2006.09.018

[53] Almendro V, Cheng YK, Randles A, Itzkovitz S, Marusyk A, et al (2014) Inference of Tumor Evolution during Chemotherapy by Computational Modeling and In Situ Analysis of Genetic and Phenotypic Cellular Diversity. Cell Rep 6: 514–527. 24462293 10.1016/j.celrep.2013.12.041 PMC3928845

[54] Altschuler SJ, Wu LF (2010) Cellular heterogeneity: when do differences make a difference? Cell 141: 559–563. 20478246 10.1016/j.cell.2010.04.033 PMC2918286

[55] Gerdes MJ, Sevinsky CJ, Sood A, Adak S, Bello MO, et al (2013) Highly multiplexed single-cell analysis of formalin-fixed, paraffin-embedded cancer tissue. Proceedings of the National Academy of Sciences of the United States of America 110: 11982–11987. 23818604 10.1073/pnas.1300136110 PMC3718135

[56] Giuliano KA, DeBiasio RL, Dunlay RT, Gough A, Volosky JM, et al (1997) High-Content Screening: A New Approach to Easing Key Bottlenecks in the Drug Discovery Process. J Biomol Screen 2: 249–259.

[57] Taylor DL, Haskins JR, Giuliano KA (2007) High content screening : a powerful approach to systems cell biology and drug discovery. Totowa, N.J.: Humana Press. xiii, 444 p. p.

[58] Haney SA, editor (2008) High Content Screening: Science, Techniques, and Applications. Hoboken: Wiley.

[59] Inglese J, editor (2006) Methods in Enzymology: Vol 414. Measuring Biological Responses with Automated Microscopy. London: Elsevier.

[60] Wang Y-l, Taylor DL, editors (1989) Flourescence Microscopy of Living Cells in Culture, Part B: Quantitaive Flourescence Microscopy-Imaging and Spectroscopy. San Diego: Academic Press. 503 p.

[61] Wang Y-l, Taylor DL, editors (1988) Flourescence Microscopy of Living Cells in Culture, Part A: Fluorescent Analogs, Labeling Cells, and Basic Microscopy. San Diego: Academic Press. 333 p.

[62] Chakravarty A, Bowman D, Ecsedy JA, Rabino C, Donovan J, et al. (2007) Developing Robust High Content Assays. High Content Screening: John Wiley & Sons, Inc. pp. 85–109.

[63] Shannon CE (1948) A Mathematical Theory of Communication. Bell System Technical Journal 27: 379–423.

[64] Rao CR (1982) Diversity and Dissimilarity Coefficients - a Unified Approach. Theor Popul Biol 21: 24–43.

[65] Schleuter D, Daufresne M, Massol F, Argillier C (2010) A User's Guide to Functional Diversity Indices. Ecol Monogr 80: 15.

[66] Lilliefors HW (1967) On the Kolmogorov-Smirnov Test for Normality with Mean and Variance Unknown. J Am Stat Assoc 62: 399–402.

[67] Razali NM, Wah YB (2011) Power Comparisons of Shapiro-Wilk, Kolmogorov-Smirnov, Lilliefors and Anderson-Darling Tests. Journal of Statistical Modeling and Analytics 2: 13.

[68] Ong LD, LeClare PC (1968) The Kolmogorov-Smirnov test for the log-normality of sample cumulative frequency distributions. Health Phys 14: 376. 5643370

[69] Young IT (1977) Proof without prejudice: use of the Kolmogorov-Smirnov test for the analysis of histograms from flow systems and other sources. J Histochem Cytochem 25: 935–941. 894009 10.1177/25.7.894009

[70] Huang S (2010) Statistical issues in subpopulation analysis of high content imaging data. J Comput Biol 17: 879–894. 20632869 10.1089/cmb.2009.0071

[71] Ng AY, Rajapakse JC, Welsch RE, Matsudaira PT, Horodincu V, et al (2010) A cell profiling framework for modeling drug responses from HCS imaging. J Biomol Screen 15: 858–868. 20525958 10.1177/1087057110372256

[72] Garraway LA, Janne PA (2012) Circumventing cancer drug resistance in the era of personalized medicine. Cancer Discov 2: 214–226. 22585993 10.1158/2159-8290.CD-12-0012

[73] Sexton JZ, He Q, Forsberg LJ, Brenman JE (2010) High content screening for non-classical peroxisome proliferators. International journal of high throughput screening 2010: 127–140. 21132080 10.2147/IJHTS.S10547 PMC2995584

[74] Abraham Y, Zhang X, Parker CN (2014) Multiparametric Analysis of Screening Data: Growing Beyond the Single Dimension to Infinity and Beyond. J Biomol Screen. 10.1177/1087057114524987 24598104

[75] Marusyk A, Almendro V, Polyak K (2012) Intra-tumour heterogeneity: a looking glass for cancer? Nat Rev Cancer 12: 323–334. 22513401 10.1038/nrc3261

[76] Hanahan D, Weinberg RA (2011) Hallmarks of cancer: the next generation. Cell 144: 646–674. 21376230 10.1016/j.cell.2011.02.013

[77] Li HJ, Reinhardt F, Herschman HR, Weinberg RA (2012) Cancer-stimulated mesenchymal stem cells create a carcinoma stem cell niche via prostaglandin E2 signaling. Cancer discovery 2: 840–855. 22763855 10.1158/2159-8290.CD-12-0101 PMC3833451

[78] Johnston PA, Grandis JR (2011) STAT3 SIGNALING: Anticancer Strategies and Challenges. Mol Interv 11: 18–26. 21441118 10.1124/mi.11.1.4 PMC3063716

[79] Kamakura S, Oishi K, Yoshimatsu T, Nakafuku M, Masuyama N, et al (2004) Hes binding to STAT3 mediates crosstalk between Notch and JAK-STAT signalling. Nat Cell Biol 6: 547–554. 15156153 10.1038/ncb1138

[80] Raj A, van Oudenaarden A (2008) Nature, nurture, or chance: stochastic gene expression and its consequences. Cell 135: 216–226. 18957198 10.1016/j.cell.2008.09.050 PMC3118044

[81] Bollrath J, Greten FR (2009) IKK/NF-kappaB and STAT3 pathways: central signalling hubs in inflammation-mediated tumour promotion and metastasis. EMBO Rep 10: 1314–1319. 19893576 10.1038/embor.2009.243 PMC2799209

[82] Vogt PK, Hart JR (2011) PI3K and STAT3: a new alliance. Cancer Discov 1: 481–486. 22348200 10.1158/2159-8290.CD-11-0218 PMC3279943

[83] Leslie K, Gao SP, Berishaj M, Podsypanina K, Ho H, et al (2010) Differential interleukin-6/Stat3 signaling as a function of cellular context mediates Ras-induced transformation. Breast Cancer Res 12: R80. 20929542 10.1186/bcr2725 PMC3096973

[84] Engelman JA, Janne PA (2008) Mechanisms of acquired resistance to epidermal growth factor receptor tyrosine kinase inhibitors in non-small cell lung cancer. Clinical cancer research : an official journal of the American Association for Cancer Research 14: 2895–2899. 18483355 10.1158/1078-0432.CCR-07-2248

[85] Johannessen CM, Boehm JS, Kim SY, Thomas SR, Wardwell L, et al (2010) COT drives resistance to RAF inhibition through MAP kinase pathway reactivation. Nature 468: 968–972. 21107320 10.1038/nature09627 PMC3058384

[86] Chandarlapaty S, Sawai A, Scaltriti M, Rodrik-Outmezguine V, Grbovic-Huezo O, et al (2011) AKT inhibition relieves feedback suppression of receptor tyrosine kinase expression and activity. Cancer Cell 19: 58–71. 21215704 10.1016/j.ccr.2010.10.031 PMC3025058

[87] Mirzoeva OK, Das D, Heiser LM, Bhattacharya S, Siwak D, et al (2009) Basal subtype and MAPK/ERK kinase (MEK)-phosphoinositide 3-kinase feedback signaling determine susceptibility of breast cancer cells to MEK inhibition. Cancer Res 69: 565–572. 19147570 10.1158/0008-5472.CAN-08-3389 PMC2737189

[88] Pritchard JR, Bruno PM, Gilbert LA, Capron KL, Lauffenburger DA, et al (2013) Defining principles of combination drug mechanisms of action. Proceedings of the National Academy of Sciences of the United States of America 110: E170–179. 23251029 10.1073/pnas.1210419110 PMC3545813

[89] Beckman RA, Schemmann GS, Yeang CH (2012) Impact of genetic dynamics and single-cell heterogeneity on development of nonstandard personalized medicine strategies for cancer. Proceedings of the National Academy of Sciences of the United States of America 109: 14586–14591. 22891318 10.1073/pnas.1203559109 PMC3437850

[90] Azam M, Latek RR, Daley GQ (2003) Mechanisms of autoinhibition and STI-571/imatinib resistance revealed by mutagenesis of BCR-ABL. Cell 112: 831–843. 12654249 10.1016/s0092-8674(03)00190-9

[91] Wood KC, Konieczkowski DJ, Johannessen CM, Boehm JS, Tamayo P, et al (2012) MicroSCALE screening reveals genetic modifiers of therapeutic response in melanoma. Sci Signal 5: rs4. 22589389 10.1126/scisignal.2002612 PMC3498828

