# Correction: Identifying and Quantifying Heterogeneity in High Content Analysis: Application of Heterogeneity Indices to Drug Discovery

**DOI:** 10.1371/journal.pone.0120468

**Published:** 2015-03-17

**Authors:** The PLOS ONE Staff

The following information is missing from the Funding section: This work was funded in part by a Pennsylvania Department of Health CURE research grant to D. L. Taylor.
