# Supplementary figures and images for: Identifying and Quantifying Heterogeneity in High Content Analysis: Application of Heterogeneity Indices to Drug Discovery
Source: PLoS One. 2014 Jul 18;9(7):e102678. doi: 10.1371/journal.pone.0102678 (PMC4103836; doi:10.1371/journal.pone.0102678)

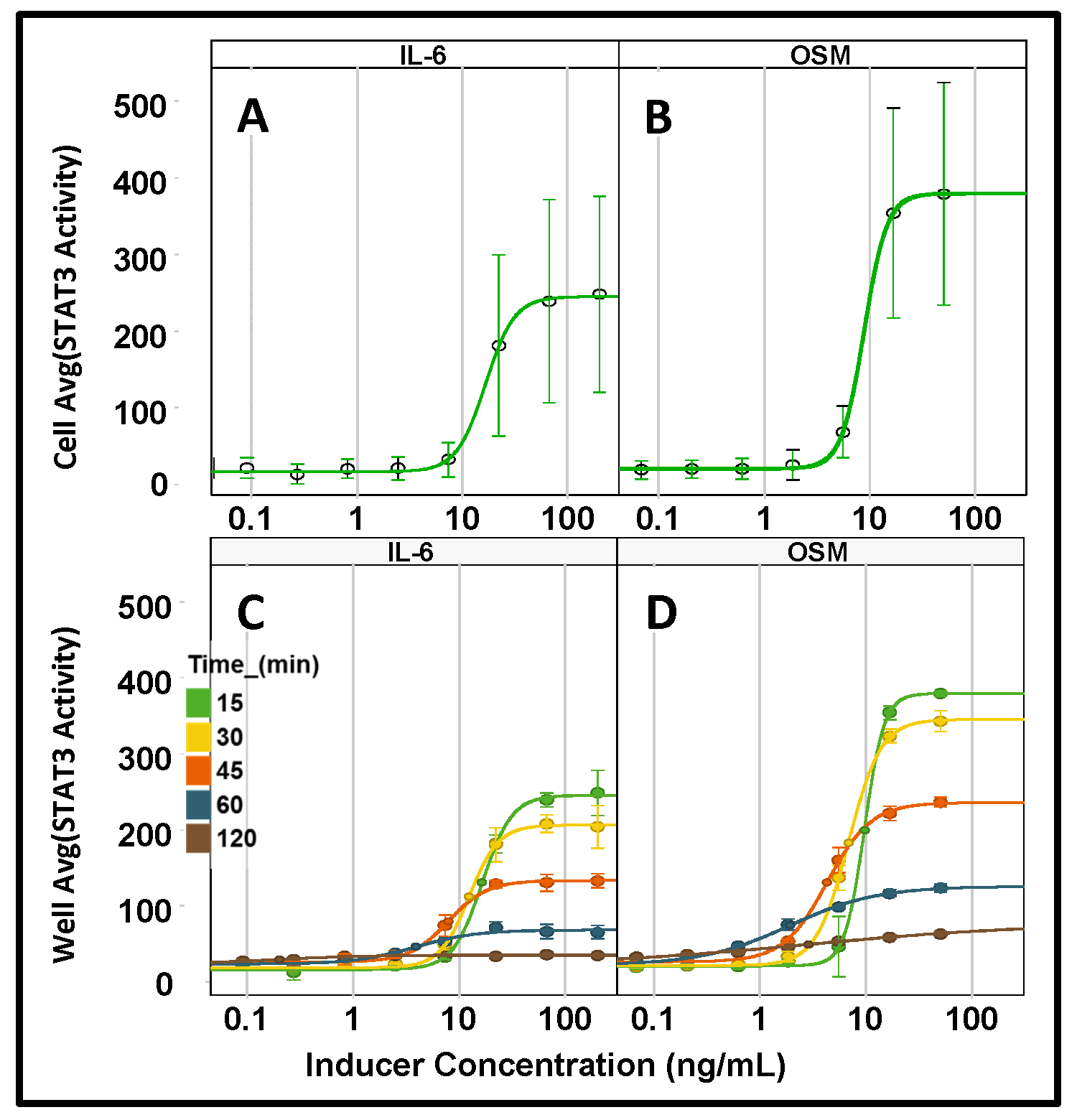

Supplement: Figure S1 — Time course and dose-response of the activation of STAT3 in Cal33 cells. A) Dose-response of IL-6 indicated by logistic regression curve fit to well average intensities in 3 replicate wells. Error bars (±1σ of the cell-by-cell intensities) indicate the high variability in the cell-to-cell STAT3 activity B) Dose-response of OSM activation of STAT3 fit as in A. Error bars (±1σ of the cellular intensities) again indicate a high degree of heterogeneity. C) Well average STAT3 activity in Cal33 cells that were exposed to the indicated concentrations of IL-6 for the times indicated by color. Error bars (±1σ of the 3 replicate wells) indicate the assay is highly reproducible despite cellular heterogeneity. D) Same as C) except cells were exposed to the indicated concentrations of OSM. Time indicated by colors. (TIF) [file pone.0102678.s001.tif]

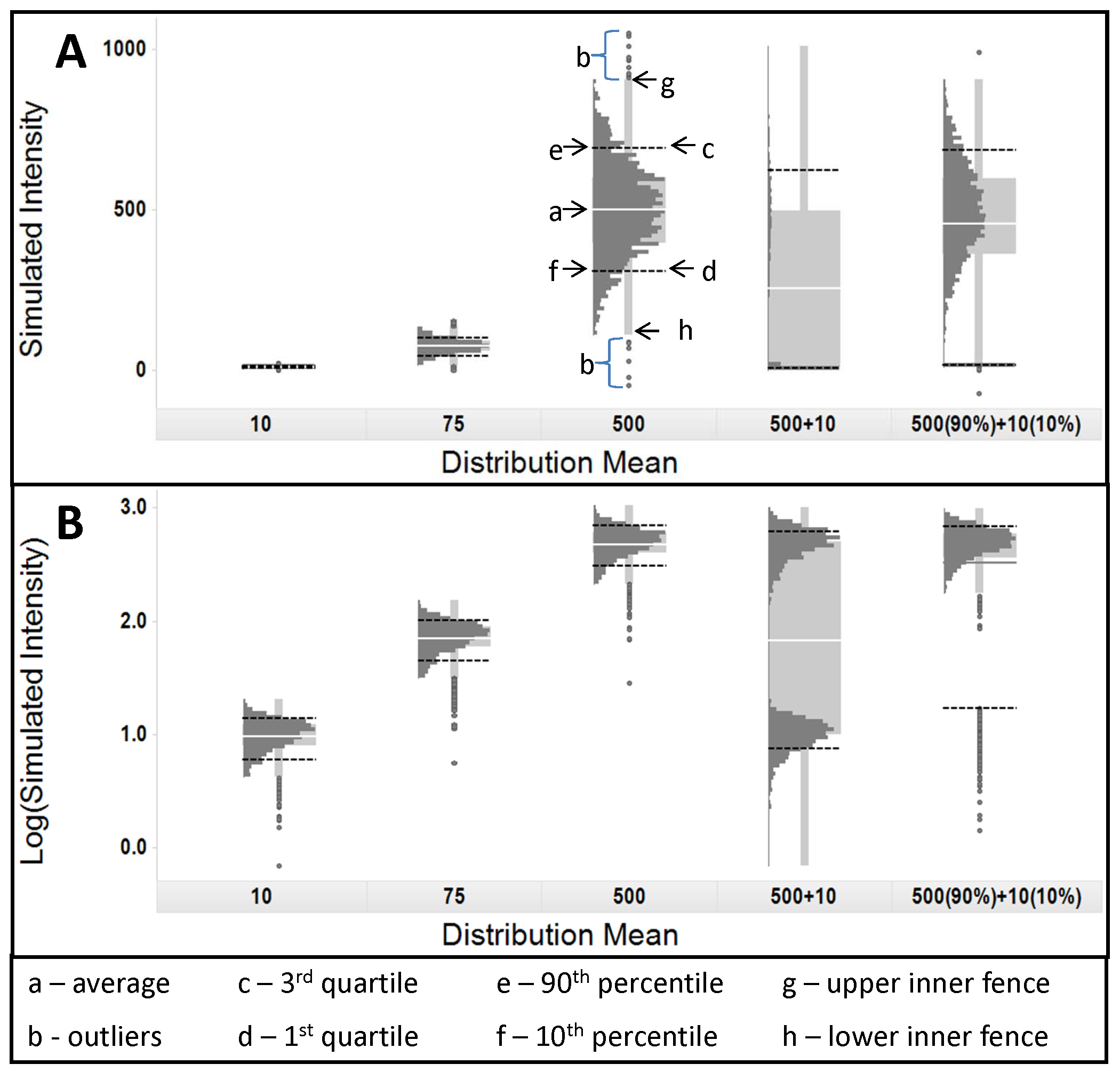

Supplement: Figure S2 — Use of Histo-Box plot for visualization of distributions. A) Histograms of simulated, normally distributed data with the indicated mean and a CV = 30% for all unimodal distributions. The 2 bimodal distributions combine the distributions with means of 500 and 10 and CV = 30%, equally weighted (‘500+10’) and with a 90∶10 split (‘500(90%)+10(10%)’). Each distribution consists of 2000 random numbers. B) The same distributions as in A, logarithmically scaled. The key defines the reference points labeled on the plot (A). (TIF) [file pone.0102678.s002.tif]

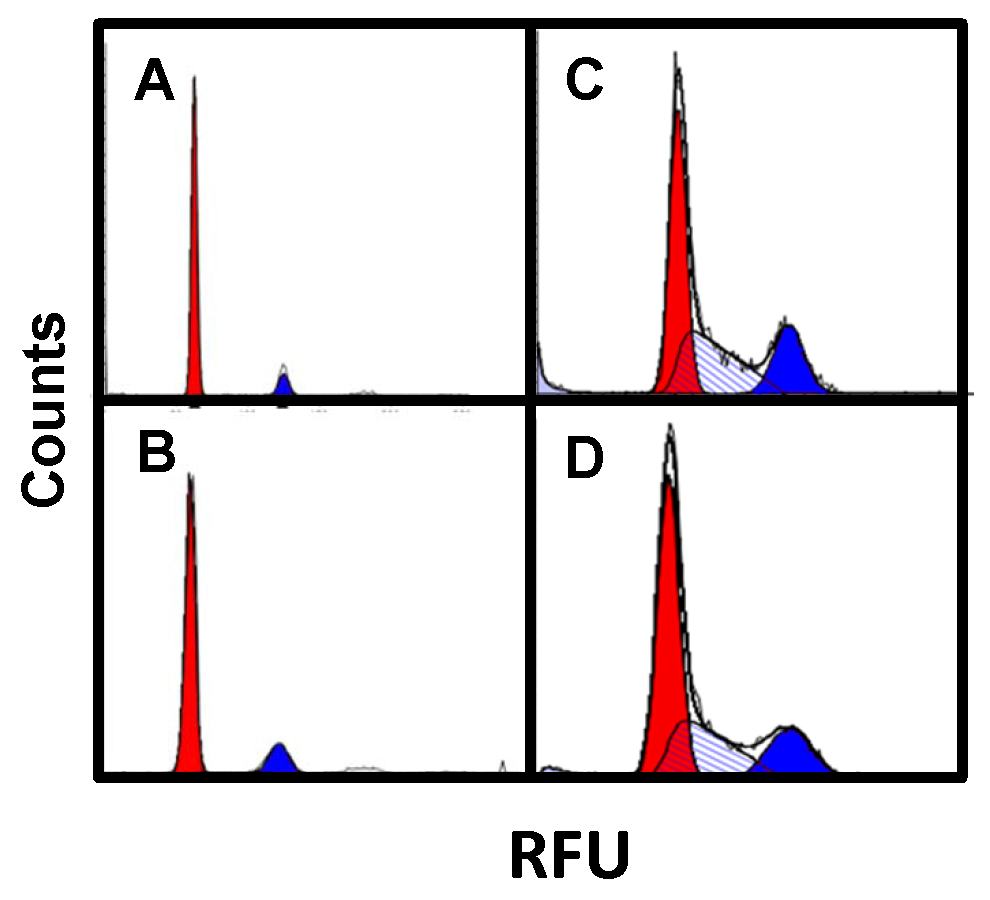

Supplement: Figure S3 — Quantitating the Reproducibility of Intensity Measurements by Flow and Imaging Cytometry. A) Histogram of the total intensity of 2 µm flow cytometry standard beads measured by flow cytometry shows peaks for single beads (red, CV = 2.8) and double beads (blue). B) Histogram of the same beads centrifuged in the wells of a 384 well microplate and imaged to measure total bead intensity also shows peaks for single beads (red, CV = 5.2) and double beads (blue). C) The histogram of total nuclear intensity in Cal33 cells fixed in suspension, labeled with Hoechst and measured by flow cytometry. Cell cycle modeling identifies 3 subpopulations, G0/G1 (red), S (hashed), and G2/M (blue). D) The histogram and cell cycle modeling of the same cells centrifuged in the wells of a 384 well microplate then imaged and analyzed for total nuclear intensity. (TIF) [file pone.0102678.s003.tif]

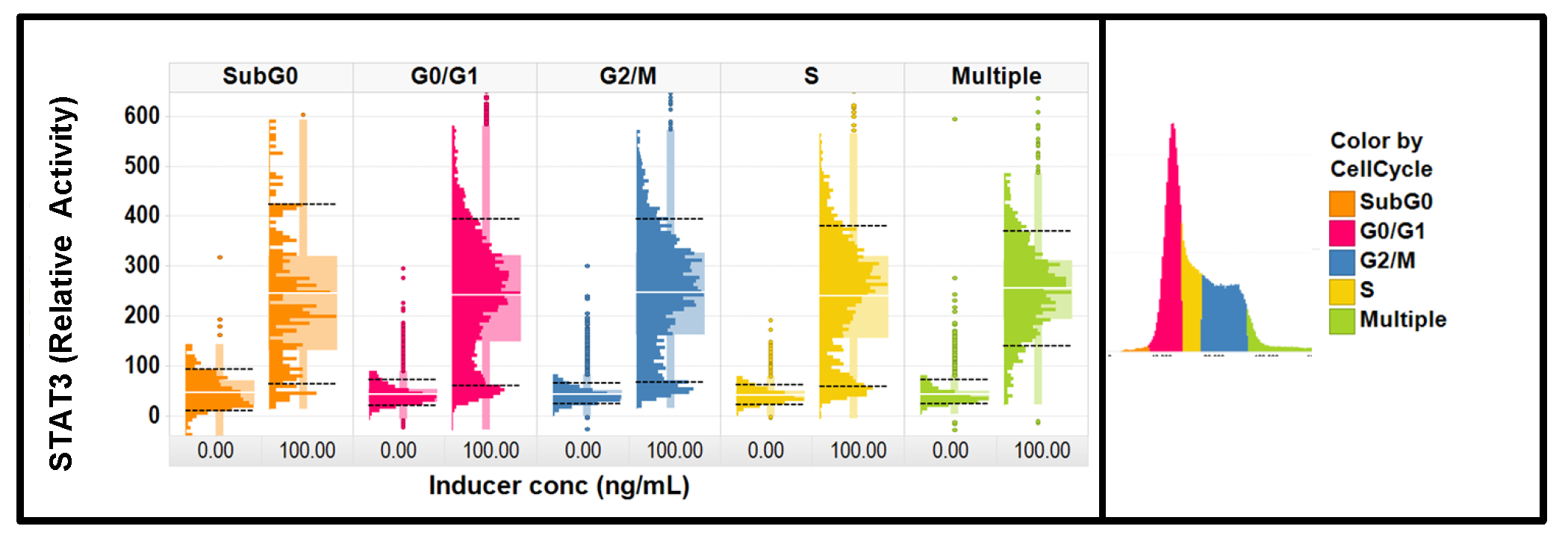

Supplement: Figure S4 — The distributions of STAT3 activation for cell cycle subpopulations. Histograms of STAT3 activation in unstimulated and IL-6 stimulated Cal33 cells (the cell cycle states identified in the legend). Inset DNA histogram of the cumulative population shows the mapping of DNA intensities to cell cycle states. (TIF) [file pone.0102678.s004.tif]

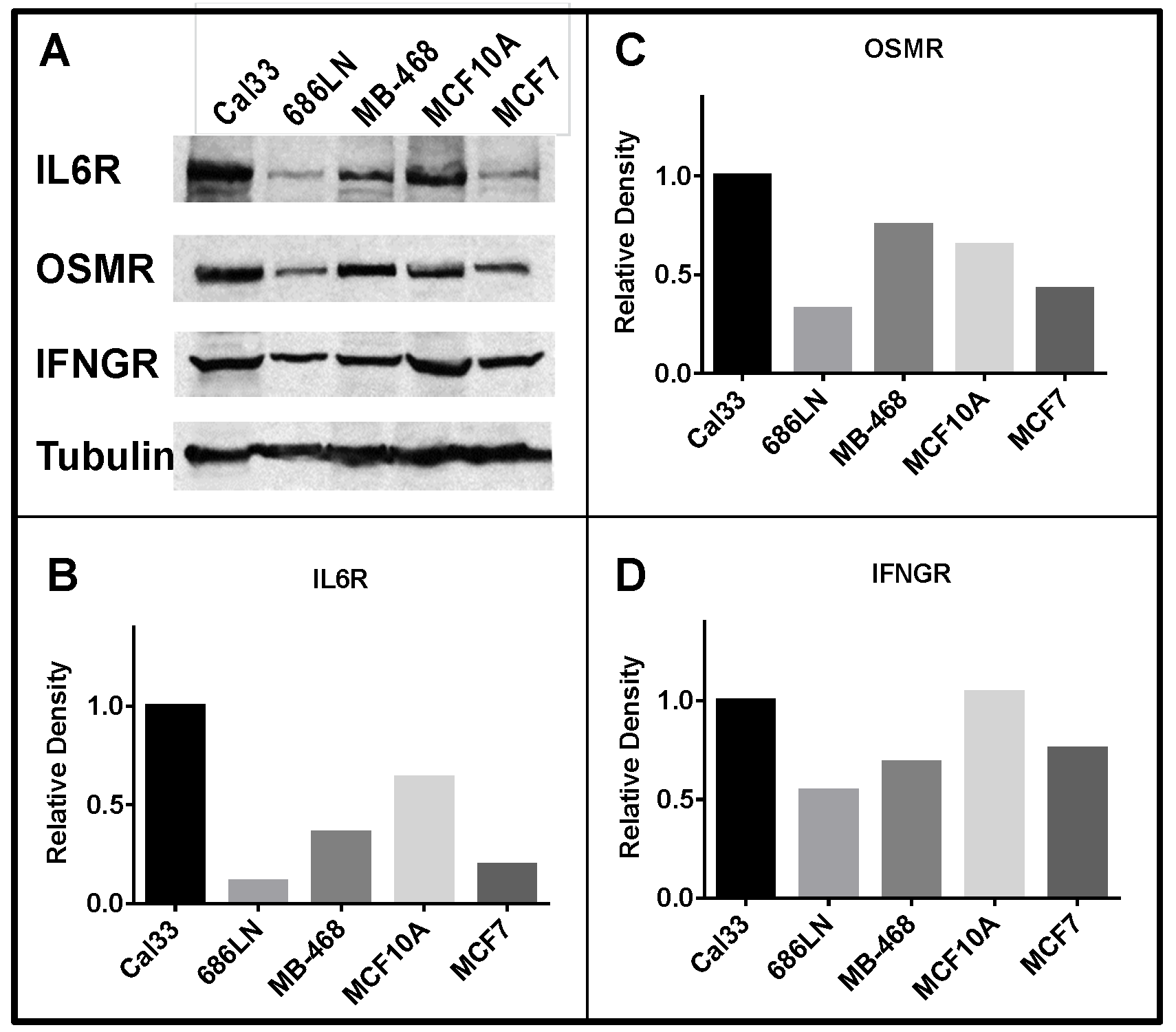

Supplement: Figure S5 — Western Blot Analysis of Receptor Expression. A) Western blots of receptor expression in five cell lines, with Tubulin as a control. B) Quantitation of total density in western blot bands for the IL-6 receptor, relative to Cal33 cells. C) Quantitation of OSM receptor expression, relative to Cal33. D) Quantitation of IFNγ receptor expression, relative to Cal33. (TIF) [file pone.0102678.s005.tif]

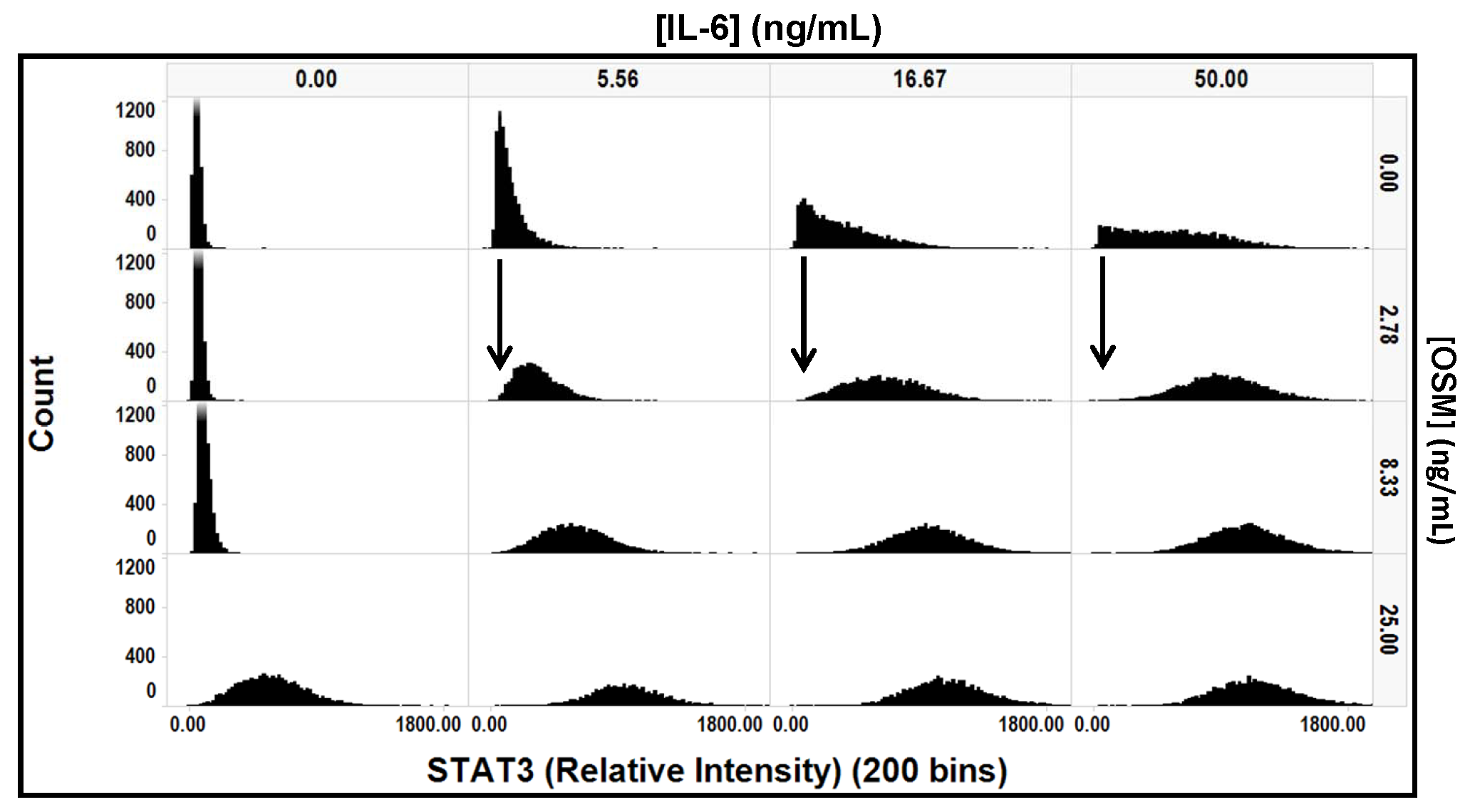

Supplement: Figure S6 — STAT3 activation by Combinations of Cytokines. To assess the interaction between the IL-6 and OSM pathways, Cal33 cells were exposed to combinations of IL-6 and OSM for 15 min. Top Row) The activation of STAT3 by IL-6 alone. Left Column) The activation of STAT3 by OSM alone. The arrows point to the location of the population non-responsive to IL-6. Note that with the addition of OSM all cells treated with IL-6 show activated STAT3. (TIF) [file pone.0102678.s006.tif]

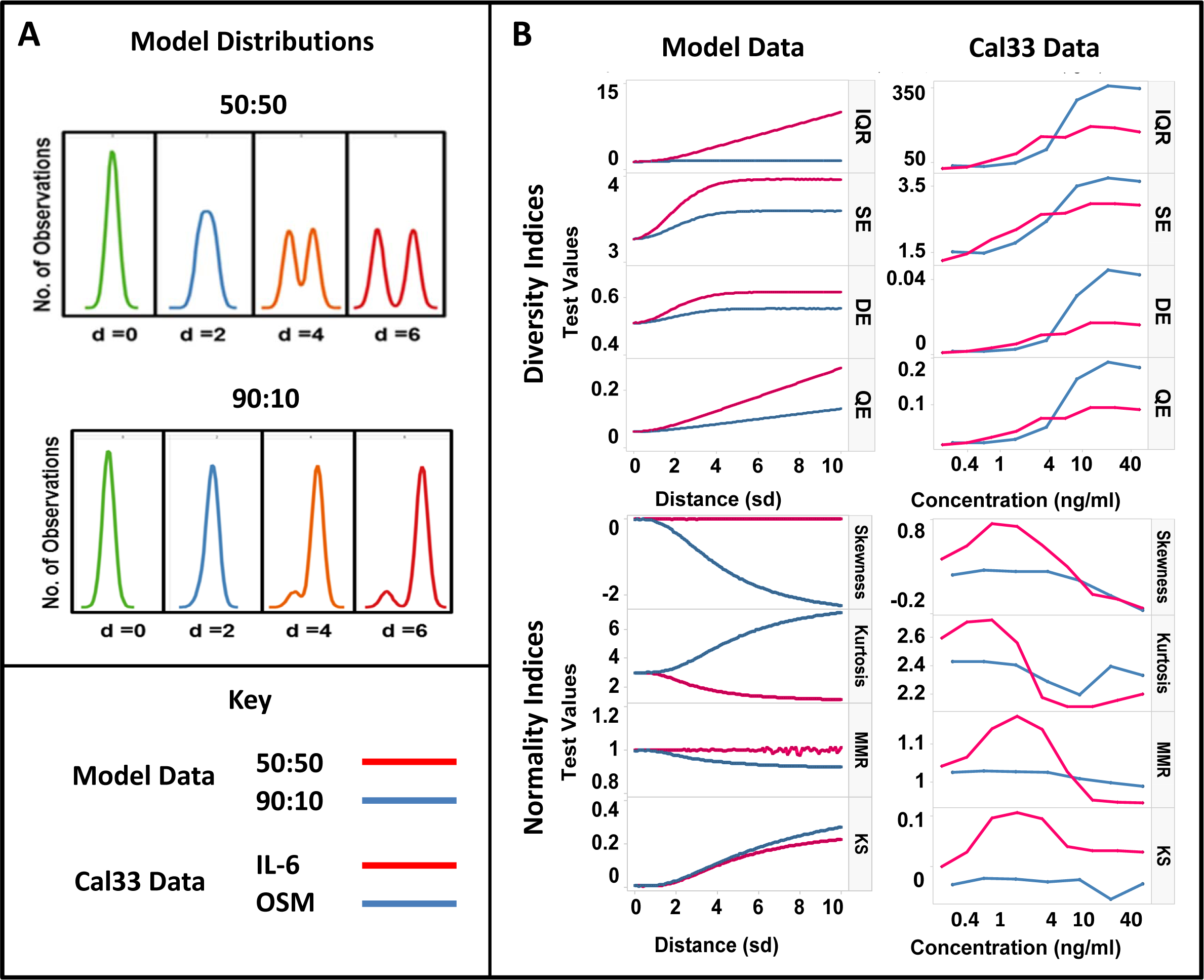

Supplement: Figure S7 — Evaluation of Potential indices of Diversity and Normality. A) Model distributions and cell data were used to evaluate the performance of selected metrics for characterizing the distributions. The 50∶50 mix consists of 2 unit normal distributions of equal population that are separated by ‘d’ standard deviations (sd). The 90∶10 mix consists of 2 unit normal distributions with 90% and 10% of the population, separated by ‘d’ standard deviations. B) Selected Diversity and Normality indices were used to evaluate the model distributions for values of ‘d’ ranging from 0–10 sd, and the Cal33 data for IL-6 and OSM stimulation. For the Cal33 data, the diversity indices all show similar response, while the model data show some key differences. The IQR (interquartile range) is not sensitive to the small 10% subpopulation, and the SE (Shannon entropy) and DE (Differential entropy, the Shannon entropy for a continuous distribution function) both plateau when the 2 populations separate. Only the QE (Quadratic entropy) shows a steady increase for both distributions. Again, the general pattern of the ‘Normality’ measures is similar for the Cal33 data, but the model data show key differences. The skewness and MMR (mean/median) are insensitive to the 50∶50 population because it is symmetric, The kurtosis and KS statistic are sensitive to the variation in both distributions, however the KS was preferred due to its direct interpretation as a measure of normality. (TIF) [file pone.0102678.s007.tif]

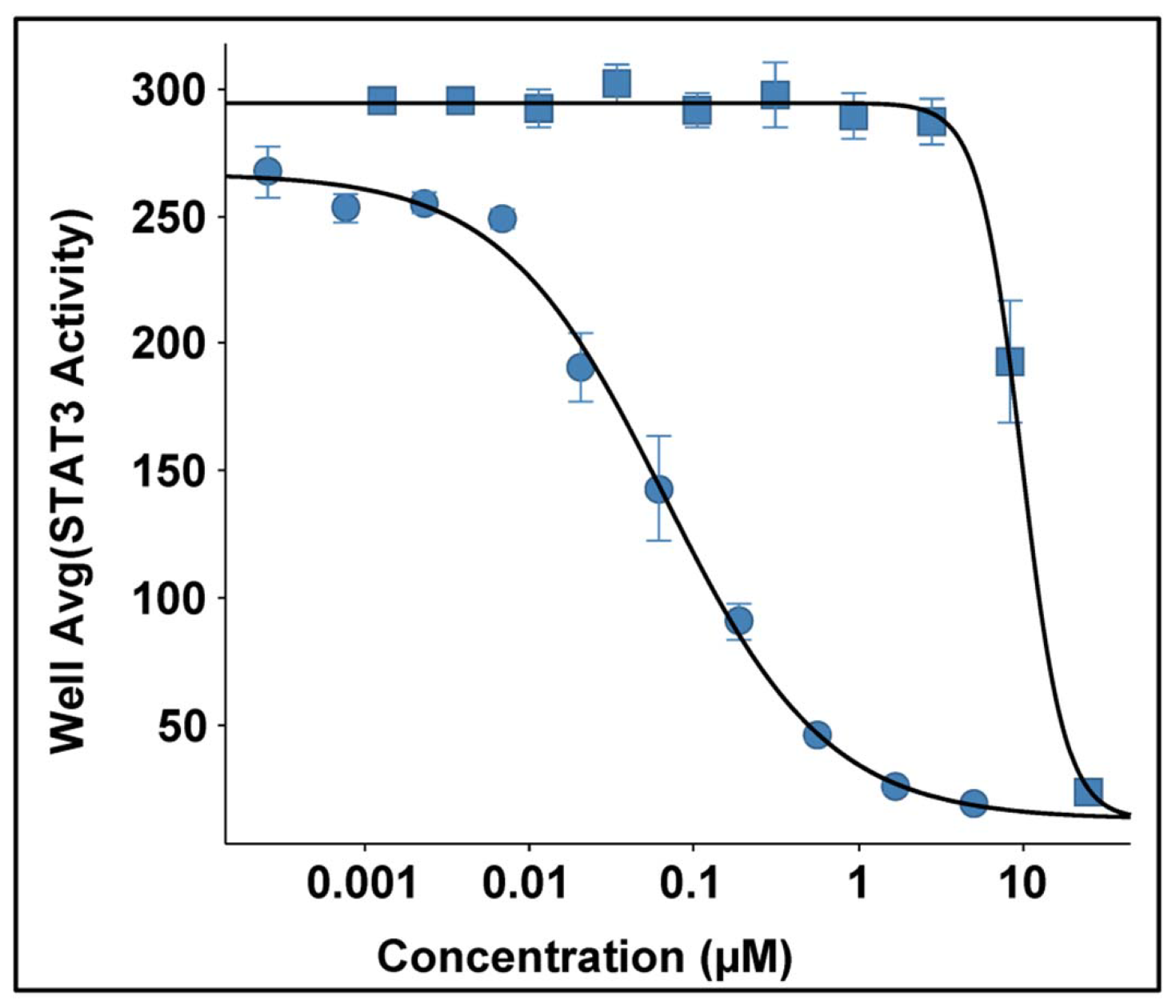

Supplement: Figure S8 — Dose dependence of inhibitors of STAT3 activation by IL-6. STAT3 activation in Cal33 cells is inhibited by Pyridone-6 (•) with an IC50 = 66 nM or STATTIC (▪) with an IC50 = 10 µM. (TIF) [file pone.0102678.s008.tif]

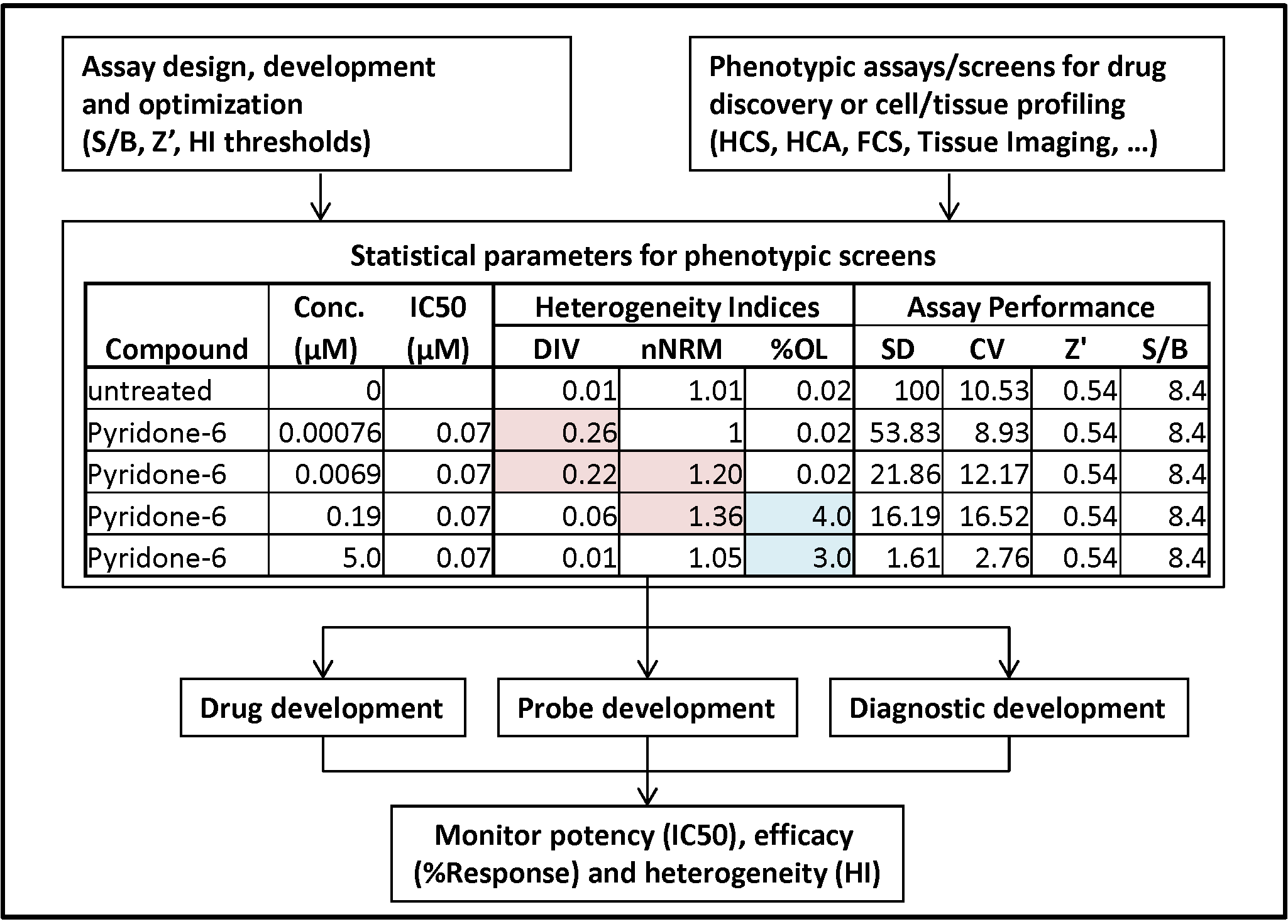

Supplement: Figure S9 — Integrating heterogeneity analysis into phenotypic screening. Heterogeneity indices are evaluated during assay development and thresholds determined based on the goals of the project. For drug discovery and cell/tissue profiling programs that encounter phenotypic heterogeneity, HCS images are analyzed to generate the features, statistical parameters and HI's. For samples or treatments with low Diversity (DIV) or a normal distribution (low nNRM) standard statistics can be used. A well or sample with a high HI and high nNRM or high %OL would require more detailed analysis of the heterogeneity. If the observed heterogeneity is biologically important in the context of the project, further experiments aimed at understanding its mechanism may lead to discovery of new targets or diagnostic biomarkers. For pyridone-6, the DIV index for concentrations below 5 µM indicates a high degree of heterogeneity (HI>0.03 from Figure 5) which is further characterized as macro-heterogeneity since the nNRM indices are >0.5. At 5 µM the DIV indicates a homogeneous population with low heterogeneity. In all cases the %OL is below the HI threshold in Figure 5. The high heterogeneity indices suggest further studies are needed to understand the activity of pyridone-6 on these cells. (TIF) [file pone.0102678.s009.tif]
