# Supplementary material for: Identifying and Quantifying Heterogeneity in High Content Analysis: Application of Heterogeneity Indices to Drug Discovery
Source: PLoS One. 2014 Jul 18;9(7):e102678. doi: 10.1371/journal.pone.0102678 (PMC4103836; doi:10.1371/journal.pone.0102678)
Supplement: Table S1 — Reproducibility of Intensity Measures. Flow cytometry standard beads and Cal33 cells were used to quantify the reproducibility of imaging intensity measurements on cells and cell sized objects. Samples of beads or cells were split and run on either the ArrayScan HCA system or a flow cytometer for reference. For beads, Ratio is (mean doublet total intensity)/(mean singlet total intensity). For Cal33 cells, Ratio is (mean G2/M total nuclear intensity)/(mean G0/G1 total nuclear intensity). (DOCX) [file pone.0102678.s012.docx]

# Table S1. Reproducibility of Intensity Measures

| **Method** | **Sample** | **CV(%)** | **Ratio*** | **S(%)** | **N** |
| --- | --- | --- | --- | --- | --- |
| **BD Spec** | 2 µm Beads | 2.3 | - | - | - |
| **Flow** | 2 µm Beads | 2.8 | 2.0 | - | 8,681 |
| **Imaging** | 2 µm Beads | 5.2 | 2.0 | - | 13,156 |
| **Flow** | Cal33 Cells | 6.2 | 1.8 | 31 | 9,522 |
| **Imaging** | Cal33 Cells | 8.0 | 1.9 | 28 | 10,814 |

# * For beads, Ratio is:

# (mean doublet total intensity)/ (mean singlet total intensity).

# For Cal33 cells, Ratio is:

# (mean G2/M total nuclear intensity)/ (mean G0/G1 total nuclear intensity)
