# Supplementary material for: Identifying and Quantifying Heterogeneity in High Content Analysis: Application of Heterogeneity Indices to Drug Discovery
Source: PLoS One. 2014 Jul 18;9(7):e102678. doi: 10.1371/journal.pone.0102678 (PMC4103836; doi:10.1371/journal.pone.0102678)
Supplement: Table S2 — Power analysis of HI measures. Replicate measures on 3 different days were used to determine the number of cells required to achieve a power of 0.8 for the CV, KS and QE measures of the distributions of STAT3 activity in Cal33 cells. (DOCX) [file pone.0102678.s013.docx]

# Table S2. Power analysis of HI measures

| **Power=0.8** | **CV** | **KS** | **QE** |
| --- | --- | --- | --- |
| **Cell Sample Size** | 850 | 1100 | 900 |
